# Composition of the human milk microbiome in the GUSTO cohort is shaped by intrapartum antibiotic prophylaxis and breastfeeding exclusivity

**DOI:** 10.1128/msystems.00677-25

**Published:** 2025-09-12

**Authors:** Lisa F. Stinson, Wei Wei Pang, Alethea Rea, Doris Fok, Mei Chien Chua, Kok Hian Tan, Fabian Yap, Keith M. Godfrey, Lynette P. Shek, Johan G. Eriksson, Yap-Seng Chong, Shiao-Yng Chan, Mary Wlodek, Donna T. Geddes

**Affiliations:** 1 School of Molecular Sciences, The University of Western Australia 550240, Perth, Australia; 2 ABREAST Network 37580, Perth, Australia; 3 UWA Centre for Human Lactation Research and Translation, Perth, Australia; 4 Global Centre for Asian Women’s Health (GloW), Yong Loo Lin School of Medicine, National University of Singapore 59053, Singapore, Singapore; 5 Bia-Echo Asia Centre for Reproductive Longevity & Equality (ACRLE), Yong Loo Lin School of Medicine, National University of Singapore 37580, Singapore, Singapore; 6 Department of Obstetrics and Gynaecology, Yong Loo Lin School of Medicine, National University of Singapore 37580, Singapore, Singapore; 7 Mathematics and Statistics, Murdoch University 37579, Perth, Australia; 8 Department of Neonatology, National University Hospital, Singapore, Singapore; 9 Department of Neonatology, KK Women’s and Children’s Hospital, Singapore, Singapore; 10 Duke-NUS Medical School 121579, Singapore, Singapore; 11 Department of Maternal Fetal Medicine, KK Women’s and Children’s Hospital 303181, Singapore, Singapore; 12 Department of Paediatrics, KK Women’s and Children’s Hospital, Singapore, Singapore; 13 Lee Kong Chian School of Medicine, Nanyang Technological University, Singapore, Singapore; 14 MRC Lifecourse Epidemiology Centre, University of Southampton 47980, Southampton, United Kingdom; 15 NIHR Southampton Biomedical Research Centre, University of Southampton and University Hospital Southampton NHS Foundation Trust, Southampton, United Kingdom; 16 Department of Paediatrics, Yong Loo Lin School of Medicine, National University of Singapore and National University Health System, Singapore, Singapore; 17 Khoo Teck Puat-National University Children's Medical Institute, National University Health System 584889, Singapore, Singapore; 18 Singapore Institute for Clinical Sciences (SICS), Agency for Science, Technology and Research (A*STAR), Singapore, Singapore; 19 Department of General Practice and Primary Health Care, University of Helsinki and Helsinki University Hospital, Helsinki, Finland; 20 Folkhälsan Research Center 3812 , Helsinki, Uusimaa, Finland; 21 Department of Obstetrics & Gynaecology and Human Potential Translational Research Programme, Yong Loo Lin School of Medicine, National University of Singapore 596630 , Singapore, Singapore; 22 Department of Obstetrics and Gynaecology, The University of Melbourne 236723, Parkville, Australia; North Carolina Agricultural and Technical State University, Greensboro, North Carolina, USA

**Keywords:** human microbiome, breast milk, 16S RNA

## Abstract

Human milk contains a low biomass microbiome, which is thought to contribute to mammary and infant health. However, the determinants of the human milk microbiome across populations are poorly understood. Here, we characterized the microbiome of 266 milk samples collected from 208 mothers at 3 weeks and 3 months postpartum from the Growing Up in Singapore Toward healthy Outcomes (GUSTO) study, a multi-ethnic Asian cohort. Full-length 16S rRNA gene sequencing showed a substantial level of inter-individual variation, as well as temporal variation, highlighting the need for broad-scale longitudinal sampling in this field. Milk microbiome composition and diversity were associated with maternal, socioeconomic, and breastfeeding factors. In particular, intrapartum antibiotic prophylaxis and breastfeeding exclusivity modulated milk bacterial composition and diversity, even at 3 months postpartum. These results highlight the dynamic nature of the human milk microbiome and its relationship with clinical and breastfeeding factors, with potential implications for infant health.

**IMPORTANCE:**

Human milk exposes infants to a constant source of maternal bacteria that may influence the development of the infant immune system and gut microbiome. However, compared to other body niches, the human milk microbiome is relatively under-studied, and there is limited consensus on the factors driving variance in these bacterial communities. In this study, we performed in-depth microbiome profiling of milk samples from 208 mothers in a diverse Asian population, finding a high level of variation between individuals and over time. We found that factors such as delivery-related antibiotics, breastfeeding practices, and maternal lifestyle can influence which bacteria are present in milk. These findings suggest that the milk microbiome is not static, but dynamic and shaped by both medical and social factors. Understanding what drives variance in the milk microbiome could help inform strategies to support maternal and infant health, especially in the critical early months of life when microbial exposures can have long-term effects.

## INTRODUCTION

Consumption of human milk has significant impacts on infant and life-long health (1). Human milk provides a unique and complex mixture of nutrients and bioactive compounds, which promote optimal growth and support the development and maturation of infant physiological systems (2). Breastfeeding has been associated with a reduced risk of infectious diseases, such as diarrheal diseases, pneumonia, and meningitis, as well as chronic diseases, including obesity, diabetes, and asthma (3–9).

The human milk microbiota contribute to infant health by exposing infants to viable and nonviable microbes (10), together with factors that modulate the microbiome such as immunoglobulin A (IgA), human milk oligosaccharides (HMOs), and antibacterial proteins. A number of bacterial strains, particularly those of the genus *Bifidobacterium*, have been shown to be vertically transmitted from mothers to infants via human milk (11–14). Importantly, variation within the milk microbiome has been linked to various infant health outcomes, including allergies (15, 16) and body composition (17). It is therefore important to understand the composition of the milk microbiome to better elucidate its role in the mother-milk-infant triad (18).

Differences in human milk microbiome composition have been attributed to various maternal, infant, social, dietary, and environmental factors (19–25). Ethnicity and geographical location have both been demonstrated to influence variation in the milk microbiome (19, 26, 27). Therefore, it is important to characterize the milk microbiome across diverse populations. To date, no such studies have been performed in a multiethnic Singaporean cohort. The Growing Up in Singapore Towards healthy Outcomes (GUSTO) cohort is a deeply phenotyped birth cohort designed to investigate the influence of pregnancy and early childhood conditions on subsequent health and development (28). In the present study, we describe the maternal, infant, and breastfeeding factors that shape the milk microbiome in mothers from the GUSTO cohort.

## MATERIALS AND METHODS

### Study population

This study was performed using data and samples from women participating in the GUSTO cohort study (28). Pregnant women were recruited during the first trimester of pregnancy from KK Women’s and Children’s Hospital (KKH) or National University Hospital (NUH) in Singapore between June 2009 and September 2010. Inclusion criteria were being 18 to 46 years of age, belonging to one of the three main ethnic groups of Singapore (Chinese, Malay, or Indian), and being either a citizen or a permanent resident of Singapore. Exclusion criteria included type 1 diabetes mellitus or treatment with chemotherapy or psychotropic drugs. All participants provided written informed consent, and the study was approved by the National Healthcare Group Domain Specific Review Board and the SingHealth Centralised Institutional Review Board. For this study, we included mothers who had provided a milk sample at 3 weeks or 3 months postpartum (*n* = 244). A total of 315 samples were available, 202 collected at 3 weeks postpartum, and 113 collected at 3 months postpartum. Eight participants were excluded from the data set due to reported mastitis, and 12 were excluded due to preterm delivery.

### Metadata collection

Data regarding participant educational attainment, maternal age, and ethnicity were collected during the first trimester of pregnancy through interviewer-administered questionnaires. Maternal pre-pregnancy BMI (kg/m^2^) was derived from pre-pregnancy weight reported by the women at 11–14 weeks gestation and maternal height measured at 26–28 weeks gestation (using SECA 213 Stadiometer). BMI category definitions were based on WHO Asian BMI categories (29). At 26–28 weeks’ gestation, women reported whether they had any previous breastfeeding experience. Information on parity, mode of delivery, child sex, and birth weight was extracted from medical records. At the 3 week postnatal visit conducted at home by trained research coordinators, mothers provided information on their in-hospital breastfeeding experiences, including any skin-to-skin soon after delivery and whether the baby was breastfed within hours of birth. Mothers also reported their current breastfeeding practices and were given the option to provide breastmilk samples if they were still breastfeeding (more details on milk collection in the sections below). Breastfeeding practices were captured using interviewer-administered questionnaires. At 3 weeks and 3 months postpartum, participants were asked whether the child was still breastfed, and the extent of breastfeeding was captured as “exclusive,” “predominant,” “partial” breastfeeding, or “formula feeding,” based on WHO breastfeeding definitions (30, 31). At each time point, children who were exclusively or predominantly breastfed were combined into one group, the “full breastfeeding” group (32) (herein referred to as the “exclusive breastfeeding”).

### Sample collection

Human milk samples (16 mL) were collected at 3 weeks or 3 months postpartum. On the day of the home visit, mothers were asked not to empty one breast for at least 2 hours and to express all breast milk from that breast between 6 a.m. and 10 a.m. into a milk bottle either by hand/manual expression or by using the mother’s own breast pump. Expressed milk samples were kept in the fridge. During the home visit, breast milk was warmed to 38°C, gently mixed, and divided into 2 mL aliquots. Samples were then transported on ice back to the laboratory and stored at −80°C. Samples were shipped on dry ice to The University of Western Australia, Perth, and subsequently kept frozen at −80°C until analysis.

### Microbiome analysis

Total DNA was extracted from 1 mL aliquots of milk using the Qiagen Microbial DNA kit. Samples were randomized across extraction plates to reduce batch effects. The full-length 16S rRNA gene was amplified using the primer pair 27F/1492R, as previously described (33). Briefly, the template was amplified in 30 µL reactions, made up of 6 µL template, 0.3 µM each of the forward and reverse primers, 0.75 µL each for ArctricZymes dsDNase and DTT, and 6.6 µL of water. Amplification cycling consisted of an initial heating stage of 94°C for 3 minutes, followed by 35 cycles of 94°C for 30 seconds, 55°C for 30 seconds, and 72°C for 2 minutes. A no-template control was included to assess potential contamination introduced by the PCR reagents. PCR products were purified using Macherey-Nagel NucleoMag magnetic beads and then assessed for size and concentration using a QIAXcel automated electrophoresis system. Barcoding of primary amplicons was performed in a secondary amplification procedure. Barcoding reactions were made up of 12.5 µL AccuStart II PCR ToughMix, 20 µM each of the forward and reverse uni-tagged barcoding primers, 4 ng of the template, and 5.5 µL of water. The same cycling conditions were used as in the first amplification, with five cycles performed. Barcoded samples were pooled in an equimolar concentration and purified using the QIAquick Gel Extraction Kit. SMRT bell adapters were ligated to purified amplicons and sequenced on a PacBio Sequel II at the Australian Genome Research Facility (AGRF).

Demultiplexed sequence data were processed using mothur version 1.48.0 (34). Filtering was performed to remove sequences of <1,336 bp or >1,743 bp and those containing homopolymers of >9 bases. Alignment was performed using the SILVA reference alignment database (v138) (35). Chimeric sequences were identified and removed using VSEARCH (36). Sequences were clustered into OTUs with a similarity cutoff of 0.03. Taxonomic identification of OTUs was performed using BLAST (37) with a cutoff of >98% sequence identity and 99% sequence coverage. Best-match species-level assignments are provided where available. However, for some genera (such as *Acinetobacter*), the 16S rRNA gene is not polymorphic enough to differentiate between different species (38, 39). In these cases, genus-level assignment is provided, followed by a number to differentiate distinct OTUs of the same genus. Taxonomic assignments for OTUs identified in negative extraction and negative PCR controls are reported in Table S1.

### Statistical analysis

#### Bacterial diversity

Diversity analyses were performed on subsampled data. Subsampling was performed to 1,008 reads, resulting in an average coverage of 96.2%. This excluded 29 low-yield samples from 20 individuals. Details of the participants included in the analysis after subsampling are presented in Table 1.

**TABLE 1:** Characteristics of mother-infant pairs included in this study (*n* = 208)

Characteristic	Mean ± SD or *n* (%)
Maternal age at delivery (years)	31.5 ± 4.3
Maternal ethnicity	
Chinese	155 (74.5)
Malay	28 (13.5)
Indian	25 (12.0)
Maternal highest level of education	
Below university	94 (45.4)
University	113 (54.6)
Household income	
<$6,000/month	103 (52.3)
≥$6,000/month	94 (47.7)
Maternal pre-pregnancy BMI category	
Underweight (<18.5 kg/m^2^)	21 (10.8)
Normal weight (18.5–22.9 kg/m^2^)	113 (54.3)
Overweight (23–27.4 kg/m^2^)	42 (20.2)
Obese (≥27.5 kg/m^2^)	19 (9.1)
Parity at delivery	
Primiparous	100 (48.1)
Parous	108 (51.9)
Previous breastfeeding experience	101 (49.3)
Cigarette smoke exposure^*a*^	
None (cotinine undetected, never smoker with no second-hand smoke exposure)	132 (67.3)
Some (cotinine undetected, current smoker and/or exposure to second-hand smoke)	47 (24.0)
Exposed (detectable cotinine, ≥0.17 µg/L)	17 (8.7)
Pregnancy antibiotic use	96 (60.4)
Intrapartum antibiotic prophylaxis	
Total	75 (36.1)
Ampicillin	19 (9.1)
Amoxicillin-clavulanic acid	13 (6.3)
Cephalosporins	10 (4.8)
Penicillin	31 (14.9)
Delivery mode	
Vaginal	159 (76.4)
Intrapartum caesarean section	26 (12.5)
Pre-labor caesarean section	23 (11.1)
Gestational age at delivery (weeks)	39.1 ± 1.0
Infant sex	
Female	101 (48.6)
Male	107 (51.4)
Infant placed at breast immediately after delivery	150 (76.5)
Breastfeeding initiated within hours	146 (74.5)
Exclusive breastfeeding	
3 weeks	80 (52.6)
3 months	46 (46.5)

^*a*^ Plasma cotinine measured by LC-MS/MS at 26–28 weeks’ gestation with a lower limit of detection of 0.17 µg/L (40).

Alpha diversity was assessed using richness (number of OTUs) and Shannon diversity. Multivariate linear mixed-effect models were fitted for Shannon diversity and log-transformed richness, with the following explanatory variables: maternal ethnicity, maternal age at delivery, maternal highest level of education, household income, parity, pre-pregnancy BMI (using the WHO Asian BMI categories (29)), infant sex, mode of delivery, whether the infant was placed on the breast immediately after delivery, whether breastfeeding commenced within hours of delivery, pregnancy antibiotic exposure, intrapartum antibiotic exposure, cigarette smoke exposure, breastfeeding status at 3 weeks and 3 months, and previous breastfeeding experience. Modeling was performed using the lme4 R package. All models included time as an interaction and participant ID as a random effect. AIC model selection was used, and P values of < 0.05 were considered significant.

Differences in Bray-Curtis distances between samples were modeled using adonis2 in the vegan package (41) using backward model selection. P values of < 0.05 were considered significant.

#### Taxonomic composition

Composition of the milk microbiome was analyzed at the OTU level. A total of 58,909 OTUs were recovered from these samples. OTUs that made up an average relative abundance of >0.5% and were present in at least 10% of participants were included in the analysis (22 OTUs). Differential abundance analysis was performed on center log ratio (CLR)-transformed data (42). Multivariate linear mixed-effect models were fitted for each OTU using the lme4 R package. Explanatory variables, interactions, and random effects were the same as those used for alpha diversity models.

Presence/absence of *Bifidobacterium* was assessed by χ^2^ test with Yates correction.

#### Analysis of change over time

In order to assess changes in the milk microbiome over time, subset models were created, restricted to mothers who provided samples at both time points only (*n* = 166 samples from 58 mothers). This ensured that participants with samples at one time point only did not leverage the data.

## RESULTS

**Fig 1:**
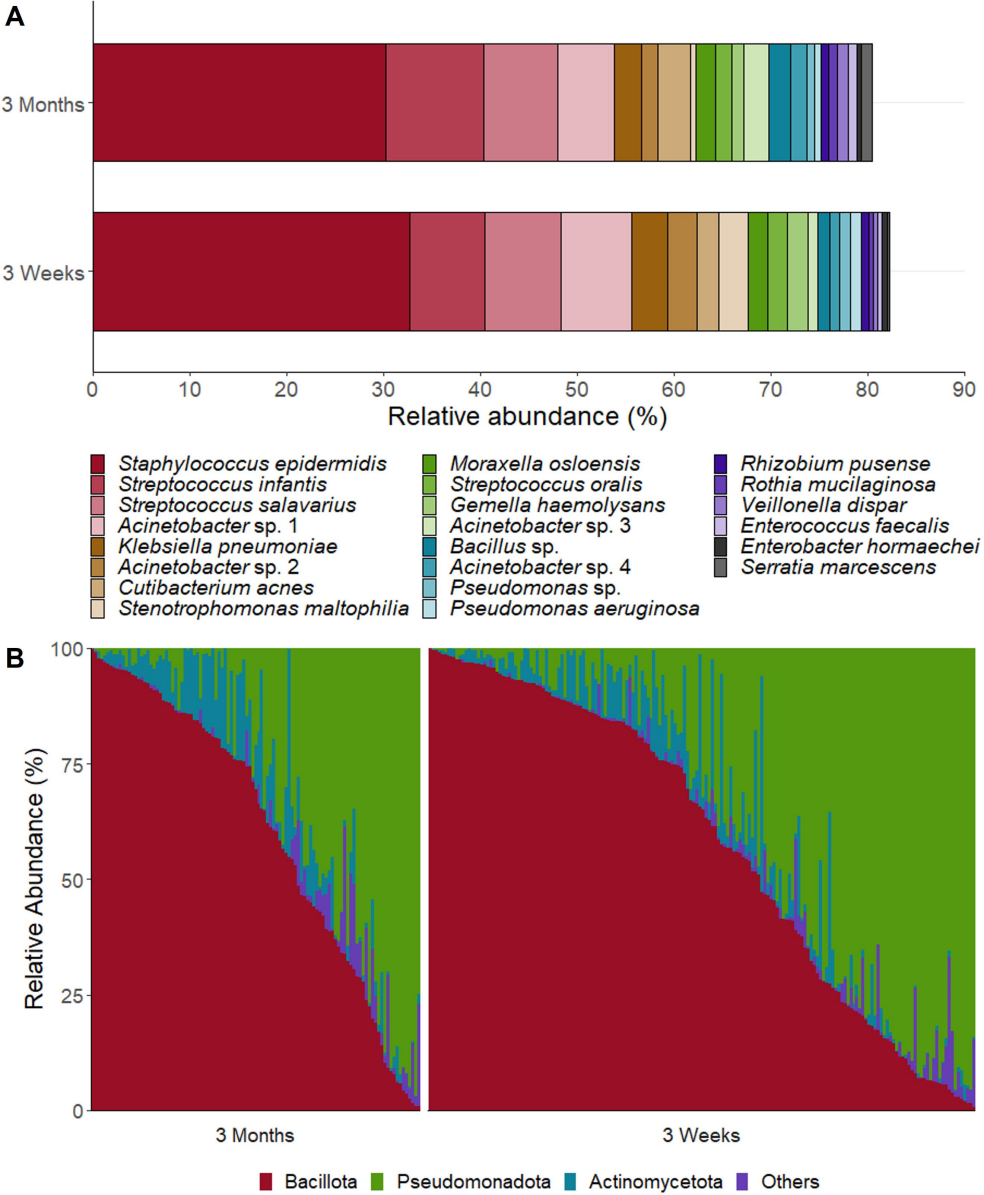
Composition of the human milk microbiome in the GUSTO cohort at 3 weeks and 3 months postpartum. (**A**) Relative abundance of top taxa (>0.5% relative abundance). (**B**) Interindividual variation in milk microbiome composition at the phylum level.

The milk microbiome of mothers in the GUSTO cohort consisted predominantly of *Staphylococcus* and *Streptococcus* species, as has been previously reported in other populations (17, 19, 25, 26, 43) (Fig. 1A). Additionally, taxa that are typical of the infant oral cavity (*Gemella, Veillonella, Rothia,* and *Strenotrophomas*), the upper respiratory tract (*Klebsiella* and *Pseudomonas*), the gut (*Enterococcus and Enterobacteria*), and the skin (*Acinetobacter* and *Cutibacterium*) were identified. This community was low in richness (56.9 ± 37.8) and evenness (Shannon diversity = 1.81 ± .68), with only 22 OTUs with a mean relative abundance of >0.5% across the cohort. We observed a high level of inter-individual variation, with samples inversely dominated by Bacillota (formerly Firmicutes) or Pseudomonadata (formerly Proteobacteria) (Fig. 1B).

### The milk microbiome becomes more rich over time

**Fig 2:**
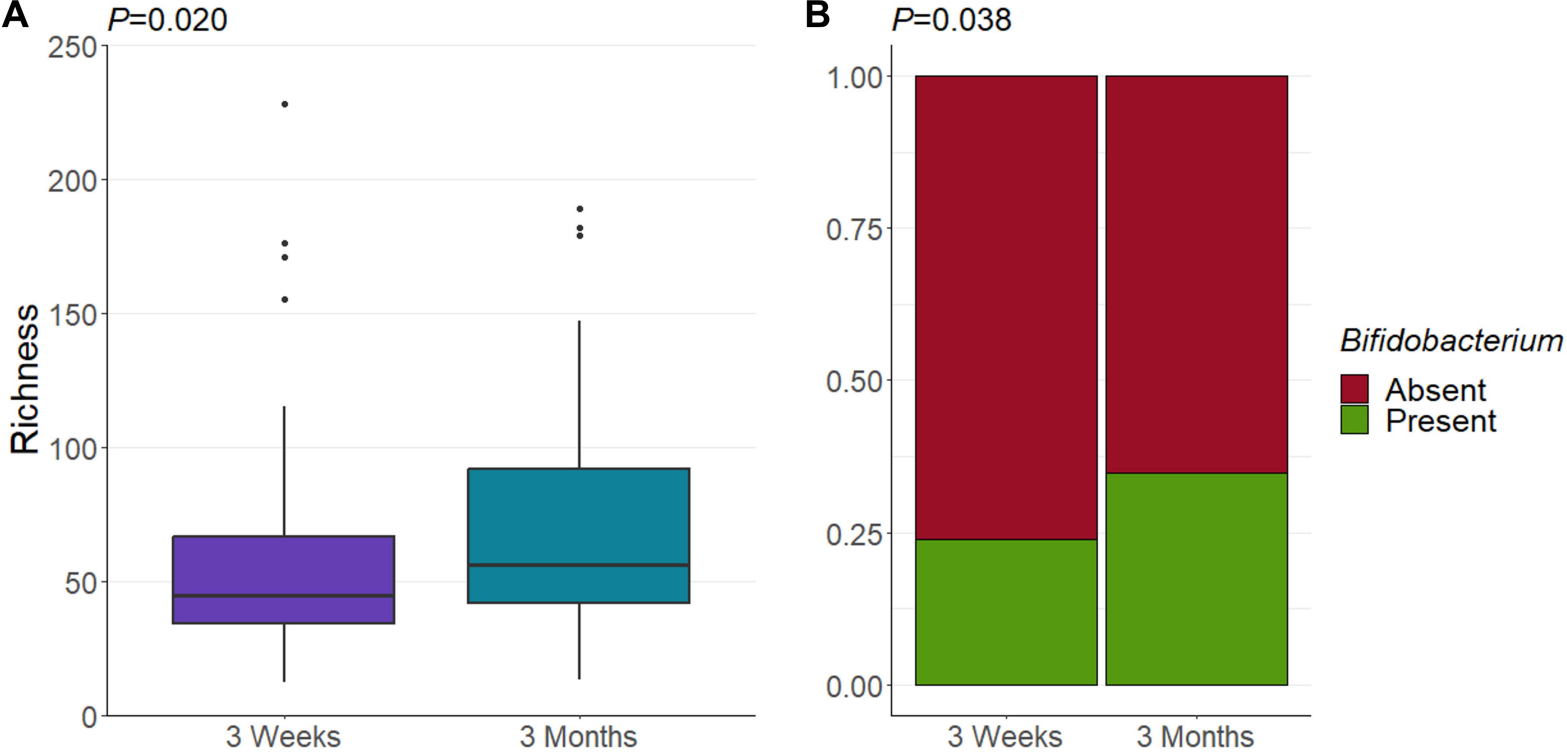
(**A**) Milk bacterial richness increases over time. *P* value derived from linear mixed-effect model. (**B**) Portion of samples that contained *Bifidobacterium* DNA. *P* value derived from the χ^2^ test.

Bacterial richness, but not Shannon diversity, increased over time (*P* = 0.020 and *P* = 0.364, respectively; Fig. 2A; Table S2). This suggests that while there is an increasing number of taxa with time postpartum, the evenness of the community remains stable. Similarly, there was no difference in bacterial community structure across time (PERMANOVA *P* = 0.374). At the OTU level, there were differences in four low-abundance OTUs over time, with less *Stenotrophomonas maltophilia* and more *Enterococcus faecalis*, *Pseudomonas* sp., and *Serratia marcescens* at 3 months compared to 3 weeks (*P* = 0.008, *P* = 0.034, *P* = 0.038, and *P* = 0.010, respectively; Table S2). However, these taxa each made up <2% relative abundance. Given that the majority of bacterial taxa vertically transmitted from milk to the infant gut are *Bifidobacterium* species (11–14, 44), we next assessed the prevalence of *Bifidobacterium* in milk over time. At 3 weeks postpartum, 23% of mothers had *Bifidobacterium* in their milk, and this increased significantly to 36% at 3 months postpartum (*P* = 0.038; Fig. 2B). While the overall abundance of this genus was low in our cohort (mean 0.38% relative abundance), in cases where it was present, it made up a relative abundance of 1.96%. Interestingly, two samples exceeded 10% relative abundance (13% and 35%). These samples each came from mothers who delivered vaginally and did not receive any antibiotics perinatally.

### Intrapartum antibiotic prophylaxis perturbs the milk microbiome to a greater extent than the delivery mode

**Fig 3:**
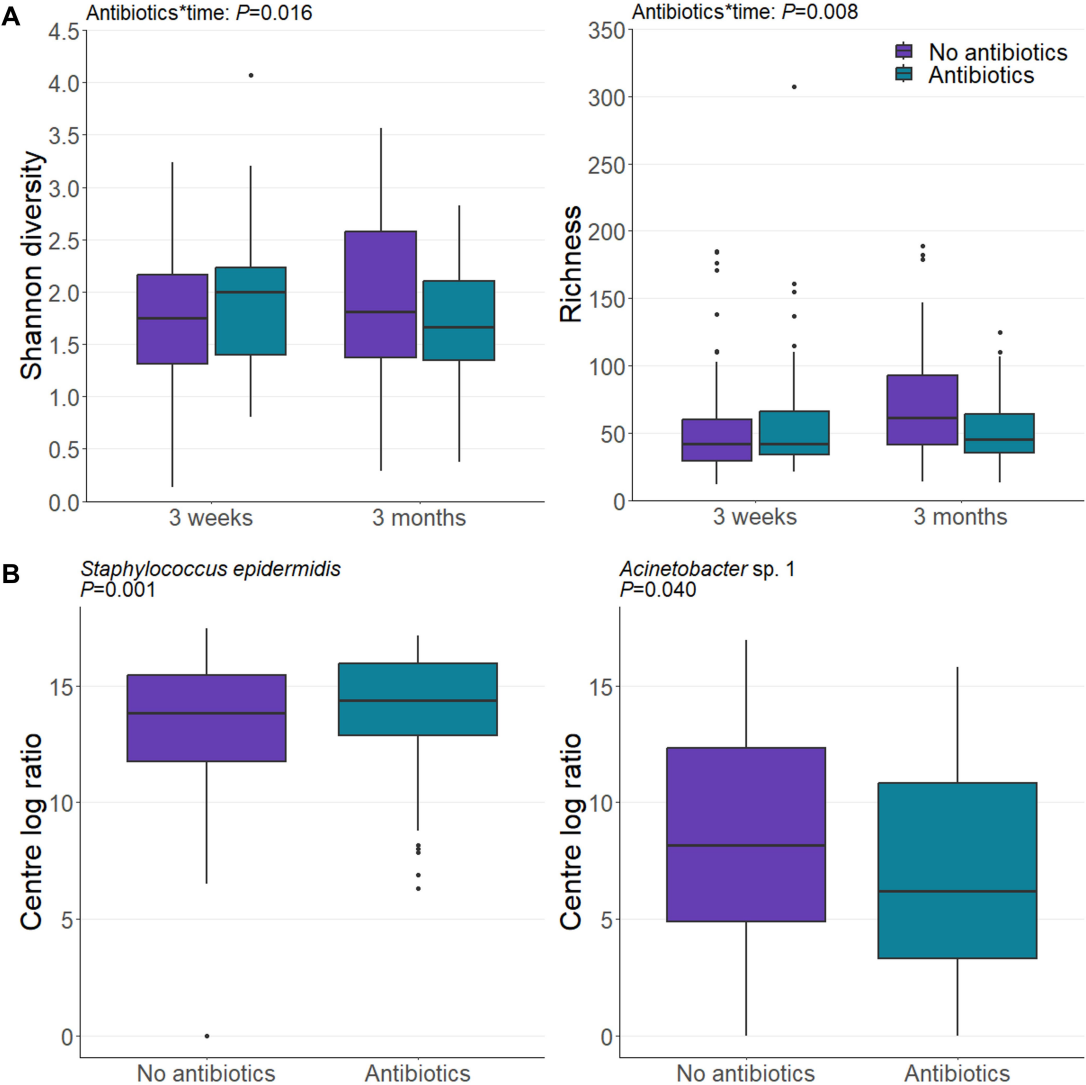
Impact of intrapartum antibiotic prophylaxis on the human milk microbiome. (**A**) A decrease in bacterial richness and Shannon diversity was observed in intrapartum antibiotic-exposed mothers (blue boxes) compared to unexposed mothers (purple boxes) at 3 months postpartum. (**B**) Intrapartum antibiotic exposure altered the abundance of seven OTUs, including the highly abundant taxa visualized here. Data are CLR-transformed abundance. *P* values are derived from linear mixed-effect models. IAP: intrapartum antibiotic prophylaxis.

Intrapartum antibiotic prophylaxis altered numerous features of the milk microbiome (Table S3). Intrapartum antibiotic use was associated with reduced richness and Shannon diversity when interacted with time (*P* = 0.008 and *P* = 0.016, respectively; Fig. 3A). Of the 22 OTUs modeled here, the relative abundance of seven was significantly shifted by intrapartum antibiotic use, including the highly abundant taxa *Staphylococcus epidermidis* and *Acinetobacter* sp. 1 (21% increase (*P* = 0.001) and 54% decrease (*P* = 0.040), respectively; Fig. 3B). Interactions between intrapartum antibiotic exposure and time were observed for four of the remaining five affected taxa, with the effect more pronounced at 3 months than at 3 weeks (Table S3).

Differential effects were observed depending on the class of antibiotic used (Table S3). Penicillin was the most widely used intrapartum antibiotic in this cohort and was administered to 41% of antibiotic-exposed mothers. Interestingly, the effects of penicillin were associated with time; at 3 months postpartum, three OTUs were associated with intrapartum penicillin use. Cephalosporins had the least impact on the milk microbiome, with only one OTU found to be associated with intrapartum cephalosporin exposure.

Exposure to antibiotics during pregnancy did not appear to affect the milk microbiome, suggesting that timing of exposure is key to the effect.

Delivery mode was also associated with the composition of the human milk microbiome, though to a lesser extent than intrapartum antibiotic prophylaxis (fewer differences detected). Given evidence of differential impacts of intrapartum and planned caesarean sections (45, 46), the delivery mode was categorized as vaginal delivery, intrapartum caesarean section delivery, and pre-labor caesarean section delivery; however, no associations were detected for pre-labor caesarean sections. Compared to mothers who delivered vaginally, those who delivered via intrapartum caesarean section had significantly higher relative abundances of *Veillonella dispar* (*P* = 0.008) and *Acinetobacter* sp. 2 (*P* = 0.006) and a significantly lower relative abundance of *Pseudomonas aeruginosa* (*P* = 0.002) at 3 months postpartum (Fig. S1). No difference in alpha or beta diversity was observed based on the delivery mode.

### The milk microbiome is shaped by breastfeeding history and exclusivity

**Fig 4:**
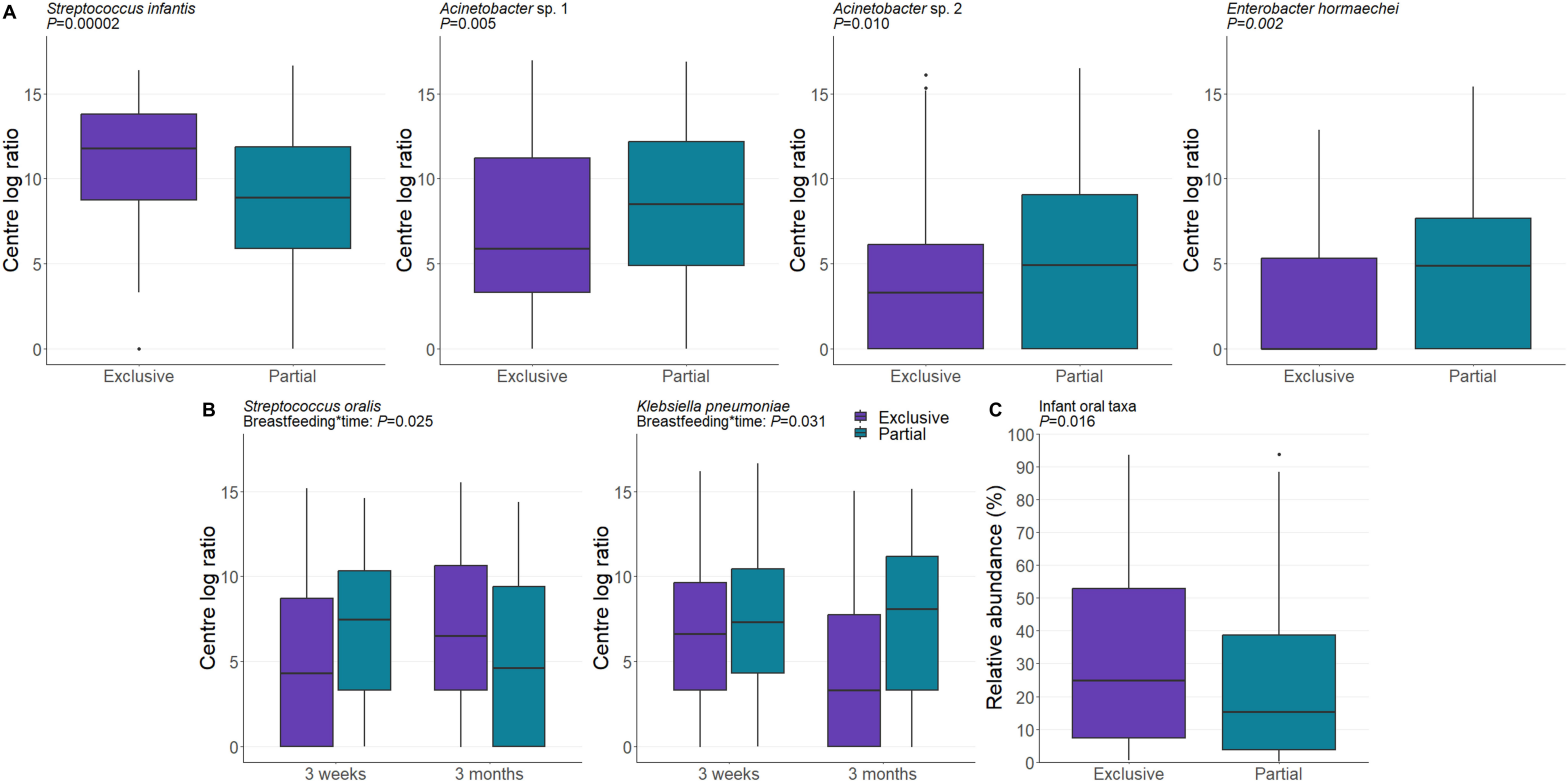
Exclusive breastfeeding modulates the human milk microbiome. (**A**) The abundances of four OTUs were significantly altered by breastfeeding exclusivity. Data are CLR-transformed abundance. (**B**) There were significant interactions between *Streptococcus oralis* and *Klebsiella pneumoniae* abundance and time. Data are CLR-transformed abundance. (**C**) The relative abundance of typical oral species was significantly higher in milk from mothers who exclusively breastfed compared to those who partially breastfed. *P* values are derived from linear mixed-effect models.

Breastfeeding exclusivity modulated the composition of the milk microbiome. Mothers who exclusively breastfed had a significantly higher relative abundance of the typical infant oral taxa *Streptococcus infantis* (*P* = 0.00002) and *Streptococcus oralis* (when interacted with time, *P* = 0.025), and significantly lower relative abundance of *Acinetobacter* sp. 1 (*P* = 0.005), *Acinetobacter* sp. 2 (*P* = 0.010)*, Enterobacter hormaechei* (*P* = 0.002), and *Klebsiella pneumoniae* (when interacted with time, *P* = 0.031) in their milk (Fig. 4A and B). These data suggest that increased interaction between the breast and the infant oral cavity during exclusive breastfeeding may increase the abundance of oral taxa in the lactating mammary gland. To test our hypothesis that increased breastfeeding drove an increase in oral taxa in the milk microbiome, we summed the relative abundances of the known oral taxa in our data set (Table S4) to create a new response variable that described the relative abundance of oral bacteria in each sample. We then modeled this response variable and found that, compared to exclusive breastfeeding, partial breastfeeding was associated with a lower total relative abundance of oral taxa in the milk microbiome (*P* = 0.016, Fig. 4C).

Similarly, mothers with previous breastfeeding experience had significantly higher relative abundances of the typical oral taxa *S. infantis* (*P* = 0.042) and *V. dispar* (when interacted with time, *P* = 0.007) compared to those with no previous breastfeeding experience. These findings appeared to be driven by breastfeeding history *per se* and not by parity, with no association between *V. dispar* and parity and a negative association between *S. infantis* and parity observed (*P* = 0.032).

Breastfeeding experiences immediately after birth also influenced the milk microbiome. If the infant was placed on the chest immediately after delivery, there was a significantly higher relative abundance of *Acinetobacter* sp. 4 and *Bacillus* sp. in the milk microbiome (*P* = 0.007 and *P* = 0.001, respectively). However, the opposite effect was observed for early initiation of breastfeeding; if breastfeeding was initiated within hours of delivery, there was a lower relative abundance of *Acinetobacter* sp. 4 in the milk microbiome (*P* = 0.010), in addition to less *Rhizobium pusense* (*P* = 0.027) and more *K. pneumoniae* (*P* = 0.007 when interacted with time). This discrepancy is difficult to explain in light of the high interrelatedness of these two variables, with 85% of those who had their infant placed immediately on their chest after delivery (*n* = 194) having also breastfed their infant within hours, while only 41% of those who did not have immediate contact with their infant (*n* = 58) breastfed within hours. However, *Acinetobacter* species have been recovered from the vaginal microbiome using both culture-dependent and -independent techniques (47). Therefore, early contact between vaginally delivered infants and the mother’s chest may introduce *Acinetobacter* to the milk microbiome, while early feeding may introduce more typical oral species.

### Maternal socioeconomic and demographic factors associated with the milk microbiome

#### Ethnicity

**Fig 5:**
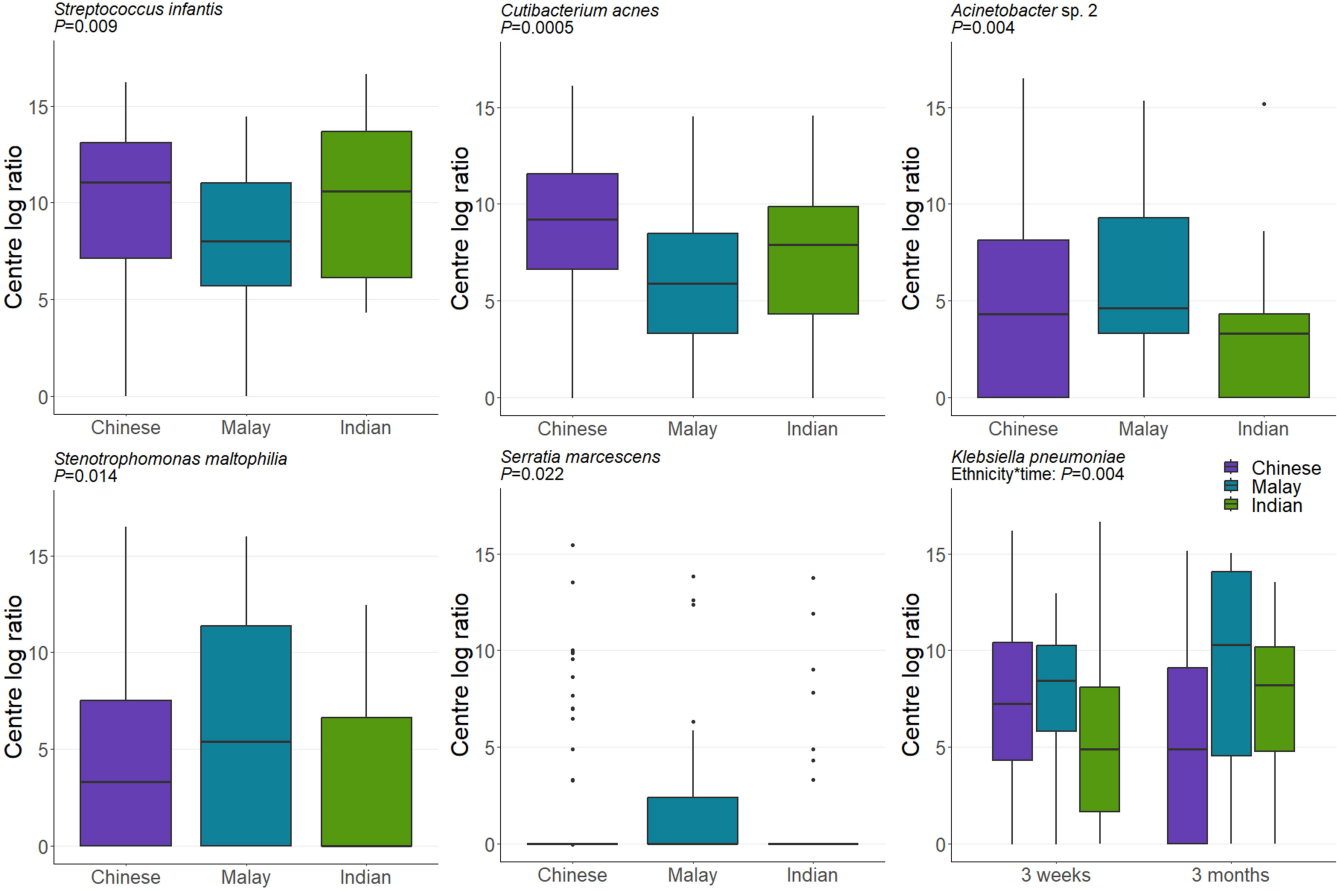
The composition of the human milk microbiome varies by maternal ethnicity. Data are CLR-transformed abundance. ANOVA *P* values derived from linear mixed-effect models are displayed.

There are three major Asian ethnic groups within the Singaporean population: Chinese, Malay, and Indian. In GUSTO, all recruited mothers had a homogeneous parental ethnic background. We identified significant associations between six milk taxa and maternal ethnicity (Fig. 5). Specifically, Malay women had less *S. infantis* and *Cutibacterium acnes* and more *S. maltophilia* and *Serratia marcescens* in their milk than Chinese women (*P* = 0.009, *P* = 0.0005, *P* = 0.014, and *P* = 0.041, respectively), while Indian women had less *Acinetobacter* sp. 2 and more *K. pneumoniae* and *S. marcescens* in their milk than Chinese women (*P* = 0.004, *P* = 0.004, and *P* = 0.033, respectively).

#### Education and income

**Fig 6:**
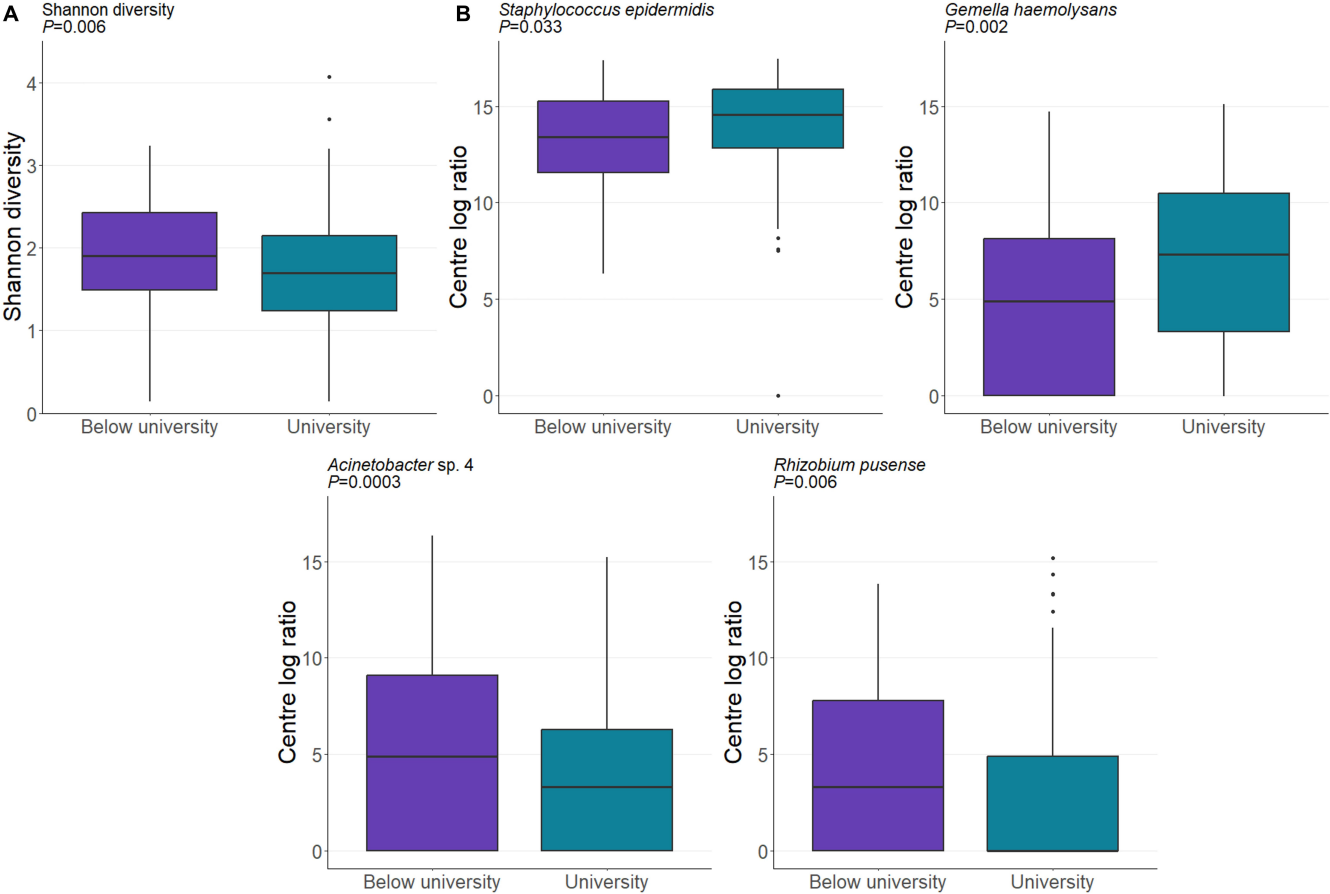
The composition of the human milk microbiome varies by the maternal level of education. (**A**) University-educated mothers had a lower milk Shannon diversity. (**B**) Maternal education level was associated with the abundances of five taxa. Data are CLR-transformed abundance. *P* values are derived from linear mixed-effect models.

The GUSTO cohort consisted of a generally highly educated population, with 55% of the mothers included in our analysis holding a university degree. University-educated mothers had a lower level of Shannon diversity in their milk (*P* = 0.006) and differential abundances of four taxa (Fig. 6). While maternal education was associated with the milk microbiome, household income was not. For this analysis, household income was divided into those who earned ≥$6,000 /month (52%) and those who earned <$6,000 /month (48%), with no significant differences in diversity or composition upon pairwise analysis.

#### Body mass index

**Fig 7:**
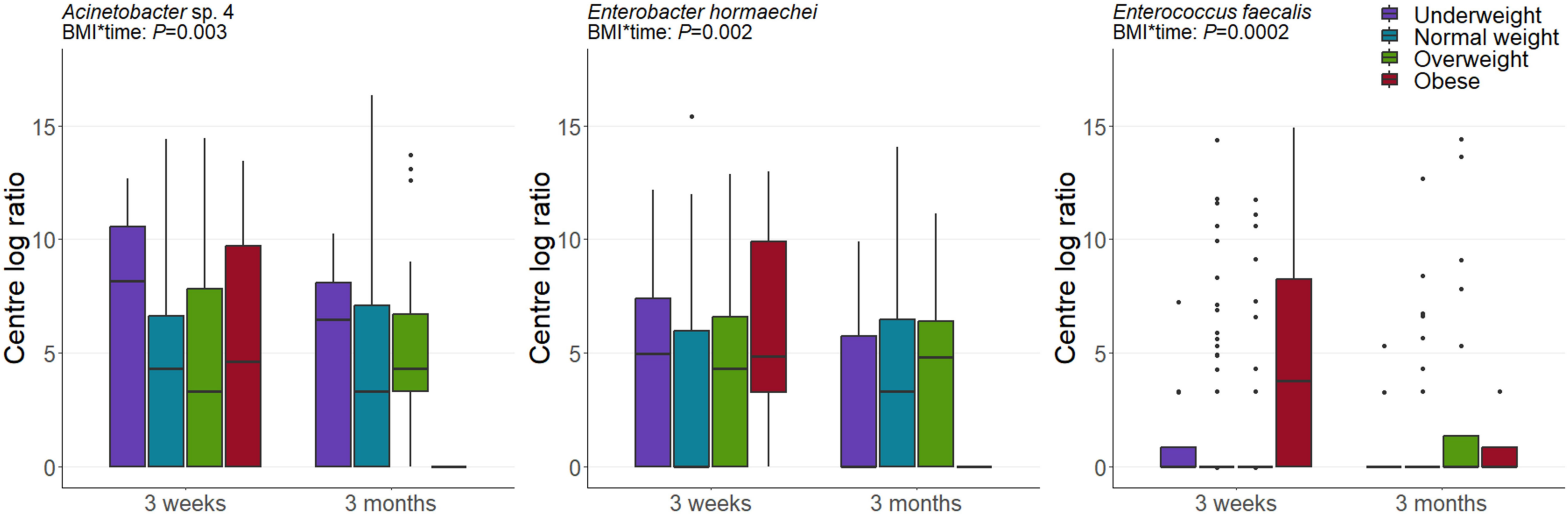
Maternal pre-pregnancy BMI is associated with the composition of the human milk microbiome. Data are CLR-transformed abundance. ANOVA *P* values derived from linear mixed-effect models are displayed.

The composition of the milk microbiome was associated with maternal pre-pregnancy BMI (Fig. 7). In particular, mothers with obesity had an altered microbiome at 3 months postpartum compared to normal weight mothers, with reduced levels of *Acinetobacter* sp. 4 and *E. hormaechei* and elevated levels of *Enterococcus faecalis* in their milk (*P* = 0.003, *P* = 0.002, and *P* = 0.0002, respectively). However, it should be noted that breastfeeding exclusivity varied across BMI categories, with exclusive breastfeeding rates of 25% for mothers with obesity and 53% for non-obese mothers.

#### Smoking, maternal age, parity, and infant sex

Maternal cigarette smoke exposure was categorized into three groups based on self-reported smoking and second-hand smoke exposure, and detection of the nicotine metabolite cotinine in maternal plasma at 26–28 weeks of pregnancy. The categories were as follows: no smoke exposure (cotinine undetected, never smoked, and no second-hand smoke), some smoke exposure (cotinine undetected, current smoker, and/or exposure to second-hand smoke), and smoke exposure (cotinine detected ≥0.17 µg/L). While smoking has previously been linked to aberrations to the gut microbiome (48, 49), the milk microbiome was largely unaffected by smoking status, with just one low abundance taxon, *E. hormaechei,* found to be elevated in the milk of smoke-exposed mothers compared to those with no smoke exposure (*P* = 0.039).

Maternal age at delivery was positively associated with six OTUs (Fig. S2). Parity and infant sex were also associated with the composition of the milk microbiome with three and four OTUs, respectively, differing according to these factors (Fig. S2 and S3).

## DISCUSSION

Here, we describe the composition and variation within the human milk microbiome of Singaporean mothers. We found that more than half of the microbiome was made up of *Streptococcus* and *Staphylococcus* species, as previously reported in studies of Chinese mothers (50–53) and mothers of other ethnicities (17, 19, 25, 26, 43). The consistency with which these two genera are found to be the dominant taxa in human milk across global populations suggests that the core microbiota of human milk are highly conserved and largely independent of ethnicity or geographical location. Our results also indicate a high abundance and diversity of *Acinetobacter* in the milk microbiome. While this genus is notorious for the nosocomial pathogens it contains, it has also been repeatedly reported as a major feature of the human milk microbiome (52, 54–58). Indeed, as a typical skin genus, it is unsurprising to find it in human milk, which consists of numerous skin taxa. Some species of *Acinetobacter* have been proposed to have a protective effect against infant allergies via dendritic cell programming (59–61), and elevated milk *Acinetobacter* abundance has been associated with protection from mastitis (57, 62). Thus, the presence of this genus in human milk may be beneficial for mothers and infants.

A strength of the present study is our use of samples taken at two time points (3 weeks and 3 months postpartum). Similar to other microbial habitats, the milk microbiome is dynamic over time (43, 58, 63), and thus cross-sectional sampling may be insufficient to holistically characterize the community. We found significant fluctuations in four low-abundance OTUs over time, in addition to an increase in the prevalence of *Bifidobacterium*. Interestingly, in our cohort, bacterial richness was significantly higher in the samples taken at 3 months compared to those taken at 3 weeks. Human milk richness has previously been shown to remain stable over time in longitudinal studies of Caucasian populations (43, 58, 64, 65). However, in Chinese, Indian, and Malay cultures, a 1-month period of maternal confinement is observed following the birth of a child. During this period, mothers and their infants remain in the home, may shower less, and adhere to a particular diet. Within the GUSTO cohort, the majority of mothers followed traditional confinement practices within the first 3 weeks postpartum (Chinese: 96.4%, Malay: 92.4%, Indian: 85.6%) (66). Our initial sample, taken at 3 weeks, fell within this period of confinement, while our second sample, taken at 3 months, occurred after confinement had ceased. Thus, the lower level of bacterial richness observed at 3 weeks postpartum may be due to reduced environmental microbial exposures resulting from confinement within the home or altered microbial patterns due to adherence to a confinement diet. We note that maternal postnatal dietary data were not available for this analysis. Given prior evidence linking maternal diet to milk microbiome composition (67–69), future studies should incorporate detailed postnatal dietary assessments alongside longitudinal milk microbiome profiling, particularly in the context of postnatal confinement. Such integrated analyses could help identify dietary factors that promote optimal early-life microbial exposures through human milk.

The strongest determinant of the milk microbiome in this cohort was intrapartum antibiotic prophylaxis. Antibiotics are powerful disruptors of the human microbiome (70, 71). While antibiotics are typically administered in multi-day courses, prophylactic intrapartum antibiotics are typically given as a single dose for caesarean deliveries, or in four hourly doses throughout labor for vaginal deliveries. Despite the short duration of administration, our data demonstrate that these drugs significantly impact the milk microbiota, reducing diversity and altering composition, with prolonged effects noticeable even at 3 months postpartum. Our results align with those of previous studies on human milk, showing a lower diversity (69, 72) and altered composition (23, 69, 72) in milk from intrapartum antibiotic-exposed mothers. Importantly, intrapartum antibiotic prophylaxis has consistently been shown to strongly disrupt the infant gut microbiome (45, 73–78), with effects reported up to 12 months of age. These results highlight the importance of studying the maternal microbiome following delivery as mothers are the primary donors of bacteria to their infants (79–81). Notably, we found that there was a delay in the impact of intrapartum antibiotics, with the effects more pronounced at 3 months than at 3 weeks. Future studies should examine the duration of the impact of intrapartum antibiotics on both the maternal and infant microbiota. Numerous attempts have been made to restore the microbiome of caesarean-delivered infants, for instance, by using probiotics, maternal fecal microbiome transplantation, or vaginal seeding (82). Vaginally delivered infants of antibiotic-exposed mothers may also benefit from these types of interventions. Interestingly, we found no effect of pregnancy antibiotic use, suggesting the timing of exposure is critical to the impact.

In addition to intrapartum antibiotic exposure, the delivery mode also influenced the milk microbiome, although to a lesser extent. As with intrapartum antibiotics, the effects were more pronounced at 3 months. Our data support previous studies demonstrating an impact of delivery mode on the milk microbiome (19, 21–23). In the present study, we found an effect of intrapartum, but not non-labor, caesarean sections. These data may suggest that the impact of delivery mode on the milk microbiome may not be mediated through the mode *per se*, but through other factors associated with intrapartum caesarean deliveries, such as prolonged labor, maternal stress, or mother-infant separation.

Breastfeeding history and exclusivity were strong determinants of the milk microbiome. Similar to previous studies, we found that breastfeeding exclusivity was associated with numerous features of the milk microbiome (83–85). Interestingly, we found a higher relative abundance of typical infant oral taxa in milk from mothers who exclusively breastfed, compared to those who partially breastfed. These data support the hypothesized bi-directional transfer of bacteria between the lactating mammary gland and the infant oral cavity. Previous studies have identified shared strains between these two habitats (86), potentially due to retrograde flow of milk during breastfeeding (87). Our data suggest that increased interaction between the breast and infant oral cavity may promote more of this kind of bacterial sharing. We also found a high abundance of the typical oral taxa *S. infantis* and *V. dispar* in milk from mothers with previous breastfeeding experience. This finding was not reflected in our parity analysis, suggesting that history of breastfeeding, rather than history of birth, drives the associations. These data are suggestive of a retained microbiome in the breast between different lactational periods. To date, no study has characterized the similarity of the milk microbiome over subsequent lactational periods; however, studies have reported the presence of a microbiota in breast tissue biopsies from nonlactating women, which include some taxa that are typical of the human milk microbiome (88–92). Thus, the milk microbiota may continue to reside in the mammary glands after lactation ceases and may influence milk composition in subsequent lactations.

Previous studies have reported variation in human milk microbiome composition based on maternal ethnicity or country of origin (19, 26, 27). In a study of Li and Han mothers residing on the Hainan island of China, ethnic differences in the microbiome of colostrum were identified (93), suggesting that ethnicity (or cultural practices) influences the milk microbiome beyond the effect of geographical location. In the GUSTO study, mothers with homogeneous parental ethnicity were included to strengthen ethnicity-based analyses. We detected variation in multiple features of the milk microbiome in Chinese, Indian, and Malay mothers, including some high-abundance taxa, such as *S. infantis* and *C. acnes*. The biological significance of these differences is unclear.

Interestingly, while household income was not associated with the milk microbiome in the present cohort, maternal education level was associated with both community evenness and composition. To our knowledge, this is the first study to identify socioeconomic status (SES) as a determinant of the human milk microbiome. However, SES is robustly associated with the gut microbiome (94–98) and may therefore also influence the milk microbiome, although current evidence for this is not strong. Importantly, maternal education, but not household income, has been associated with breastfeeding beliefs and practices in the GUSTO study (99). Similarly, breastfeeding exclusivity has repeatedly been linked to maternal education level (100–104). Thus, education-related differences in breastfeeding behaviors may explain the results in our study.

Our results also confirm that maternal pre-pregnancy obesity is associated with the composition of the milk microbiome (21, 24, 25, 58). However, all associations detected interacted with time, suggesting temporal variation in this relationship. These interactions reiterate the importance of longitudinal sampling for milk microbiome analyses. At 3 months postpartum, mothers with an obese pre-pregnancy BMI had significantly lower levels of *Acinetobacter* sp. 4 and *E. hormaechei* in their milk compared to mothers with a normal pre-pregnancy BMI. Gut *E. hormaechei* has been shown to increase dramatically in abundance following weight-loss surgery (105). *Enterobacter* harbor genes for bile salt hydrolases, which deconjugate bile acids and thereby play a role in lipid metabolism and promotion of healthy weight (106, 107). Similarly, *Acinetobacter* has previously been found to be reduced in milk from mothers with an obese BMI (58). This is in line with research showing that diet-associated weight loss drives an increase in *Acinetobacter* species in the gut (108). Thus, normal weight mothers may be expected to harbor higher levels of *Acinetobacter* and *Enterobacter* in their gut and milk than mothers with obesity. We also found an elevated level of *E. faecalis* in milk from mothers with an obese pre-pregnancy BMI, as has been reported in the gut microbiome of nonlactating individuals (109). Conversely, *Enterococcus* in both human milk and the infant gut has been shown to be inversely correlated with excessive weight gain in infants (110). However, its relationship with maternal BMI was not investigated within this study. Thus, milk *E. faecalis* levels may have differing relationships with maternal and infant BMI.

While our study was not designed to directly assess functional consequences, the observed associations with intrapartum antibiotic use, delivery mode, and maternal sociodemographic factors may have implications for infant health. Antibiotic-induced reductions in milk microbial diversity and alterations in the abundance of key taxa could influence early microbial exposure and immune development, particularly given the role of human milk as a source of pioneering microbes for the infant gut (111). Similarly, differences in the milk microbiome by delivery mode may reflect variation in maternal microbial reservoirs accessible during birth and early postpartum, potentially affecting the trajectory of infant microbiome assembly. The associations with maternal sociodemographic factors, though more subtle, may reflect underlying differences in maternal health or stress, which in turn may shape milk microbial communities.

These findings also raise the possibility of actionable interventions. For example, decisions around the use of intrapartum antibiotics could incorporate consideration of downstream microbial effects, and targeted support for exclusive breastfeeding may help promote more beneficial milk microbiome profiles. While such implications remain speculative, they underscore the importance of continued research in this area. Future studies integrating metagenomic, metabolomic, or host immune data, along with infant health outcomes, will be critical to understanding how variation in milk microbiota contributes to early-life development and to guiding clinical practice.

### Conclusions

Here, we provide the first human milk microbiome study of a multiethnic Asian cohort in Singapore. We identified a high level of interindividual variation and a composition that closely mirrors that of other populations. Intrapartum antibiotic use and breastfeeding behaviors were strongly associated with milk microbiome composition and diversity, along with multiple other maternal, environmental, and infant factors. Importantly, many of our findings interacted with time, highlighting the importance of longitudinal sampling of the milk microbiome.

## Data Availability

The data sets generated during the current study are available in the NCBI Sequence Read Archive (SRA), under BioProject ID PRJNA1108455.

## SUPPLEMENTAL MATERIAL

Supplemental Material msystems.00677-25-s0001.pdf Supplemental tables and figures.

ASM does not own the copyrights to Supplemental Material that may be linked to, or accessed through, an article. The authors have granted ASM a non-exclusive, world-wide license to publish the Supplemental Material files. Please contact the corresponding author directly for reuse.
