# Supplementary material for: Composition of the human milk microbiome in the GUSTO cohort is shaped by intrapartum antibiotic prophylaxis and breastfeeding exclusivity
Source: mSystems. 2025 Sep 12;10(10):e00677-25. doi: 10.1128/msystems.00677-25 (PMC12542769; doi:10.1128/msystems.00677-25)
Supplement: Supplemental Material — Supplemental tables and figures. [file msystems.00677-25-s0001.pdf]

Composition of the human milk microbiome in the GUSTO cohort is shaped by intrapartum antibiotic prophylaxis and breastfeeding exclusivity

Supplementary material

Table S1: Genera recovered from negative extraction (EC) and PCR (NTC) controls.

Genus	EC1	EC2	EC3	EC4	NTC1	NTC2	NTC3	NTC4
Acinetobacter	2	49	3	5	2	4	4	9
Streptococcus	1	4	2	7	2	3	6	15
Enterobacterales unclassified	0	37	0	0	0	0	0	1
Staphylococcus	2	4	2	2	0	3	3	6
Enterobacteriaceae unclassified	0	17	0	0	0	1	0	0
Gammaproteobacteria unclassified	0	14	1	0	0	0	1	1
Pseudomonas	3	7	1	1	0	0	0	3
Bacilli unclassified	1	0	0	3	0	0	2	4
Filibacter	0	0	0	10	0	0	0	0
Cutibacterium	1	1	1	1	1	1	0	3
Stenotrophomonas	1	6	0	0	1	0	0	0
Chryseobacterium	0	4	1	0	0	0	1	0
Corynebacterium	1	1	0	3	0	1	0	0
Enhydrobacter	0	2	0	3	0	0	0	1
Xanthomonadaceae unclassified	1	5	0	0	0	0	0	0
Bacillus	1	0	0	0	0	0	2	2
Delftia	1	3	0	0	1	0	0	0
Weeksellaceae unclassified	5	5	0	0	0	0	0	0
Enterobacter	1	3	0	0	0	0	0	0
Klebsiella	1	1	1	0	1	0	0	0
Lactobacillales unclassified	1	0	0	1	0	0	0	2
Sphingobacterium	0	4	0	0	0	0	0	0
Comamonadaceae unclassified	0	3	0	0	0	0	0	0
Corynebacteriaceae unclassified	0	1	0	2	0	0	0	0
Gemella	0	0	0	1	0	0	1	1
Moraxellaceae unclassified	0	1	0	1	0	0	0	1
Pseudomonadaceae unclassified	0	2	0	1	0	0	0	0
Pseudomonadales unclassified	0	3	0	0	0	0	0	0
Rothia	1	0	0	1	0	0	1	0
SN8	0	2	0	0	0	1	0	0
Sphingomonas	0	2	0	0	0	1	0	0
Sphingopyxis	0	1	0	1	0	0	0	1
Allorhizobium-Neorhizobium-Pararhizobium-Rhizobium	0	2	0	0	0	0	0	0
Bacillales unclassified	0	0	0	2	0	0	0	0
Haemophilus	0	0	0	1	0	0	1	0
Planococcaceae unclassified	0	0	0	2	0	0	0	0

Pleomorphomonas	0	2	0	0	0	0	0	0
Rhizobiaceae unclassified	0	2	0	0	0	0	0	0
Serratia	0	0	1	0	0	1	0	0
Sphingobacteriaceae unclassified	0	2	0	0	0	0	0	0
Sphingomonadaceae unclassified	0	2	0	0	0	0	1	0
Streptococcaceae unclassified	0	2	0	0	0	0	0	0
Acidovorax	0	1	0	0	0	0	0	0
Aeromonas	0	0	0	1	0	0	0	0
Alkanindiges	0	1	0	0	0	0	0	0
Azomonas	0	1	0	0	0	0	0	0
Azorhizobium	0	1	0	0	0	0	0	0
Bacillaceae unclassified	0	0	0	0	0	0	0	1
Bdellovibrio	0	1	0	0	0	0	0	0
Branchiibius	0	0	1	0	0	0	0	0
Brevibacillus	0	0	0	1	0	0	0	0
Brevundimonas	0	0	0	1	0	0	0	0
Capnocytophaga	0	0	0	0	0	0	1	0
Ciceribacter	0	1	0	0	0	0	0	0
Cronobacter	0	1	0	0	0	0	0	0
Dokdonella	0	0	1	0	0	0	0	0
Erwiniaceae unclassified	0	1	0	0	0	0	0	0
Flavobacterium	0	1	0	0	0	0	0	0
Gordonia	0	0	1	0	0	0	0	0
Granulicatella	0	0	0	0	0	0	1	0
Neisseria	0	0	0	0	0	0	1	0
Oceanobacillus	0	0	1	0	0	0	0	0
Pantoea	0	0	0	0	0	0	0	1
Paracoccus	0	0	0	0	0	0	0	1
Pasteurellaceae unclassified	0	0	0	0	0	0	1	0
Phyllobacterium	0	0	0	0	0	1	0	0
Propionibacteriaceae unclassified	0	0	0	0	0	1	0	0
SM1A02	0	0	1	0	0	0	0	0
SM2D12 unclassified	0	0	1	0	0	0	0	0
Sphingobium	0	1	0	0	0	0	0	0
Sporosarcina	0	0	0	1	0	0	0	0
Staphylococcaceae unclassified	0	1	0	1	0	0	0	0
Veillonella	0	0	0	0	0	0	0	1

Table S2: Outputs of linear mixed effects models, with the analysis restricted to mothers who provided samples at both time points (n=166 samples from 58 mothers). Models were fitted for each response variable. Significant P values are reported. ~ represents an interaction between two variables.

Response	Variable	Estimate	Standard Error	t value	P-value	ANOVA P-value
**Richness**	Household income: ≥ $6000	-0.38	0.12	-3.21	0.002	0.006
	Time: 3 Months	0.22	0.09	2.40	0.020	0.016
**Shannon diversity**	Ethnicity: Malay ~ Time: 3 Months	0.81	0.37	2.19	0.033	0.026
	Ethnicity: Indian	0.20	0.27	0.73	0.466	
	Ethnicity: Indian ~ Time: 3 Months	-0.45	0.35	-1.29	0.203	
**Otu00001 *Staphylococcus epidermidis***	Augmentin	2.39	1.08	2.20	0.032	0.027
	Time: 3 Months ~ Breastfeeding: Partial	1.69	0.82	2.07	0.043	0.039
**Otu00002 *Streptococcus salavarius***	Smoking: Some exposure	3.54	2.73	1.30	0.197	
	Smoking: Missing	7.34	3.76	1.95	0.054	
	Time: 3 Months ~ Intrapartum antibiotics	-3.58	1.58	-2.26	0.028	0.024
	Time: 3 Months ~ Breastfeeding: Partial	-2.82	1.23	-2.30	0.025	0.022
	Time: 3 Months ~ Penicillin	5.35	2.03	2.64	0.011	0.008
	Time: 3 Months ~ Smoking: Some exposure	-6.15	2.79	-2.20	0.032	
	Time: 3 Months ~ Smoking: No exposure	-3.29	2.52	-1.31	0.198	0.005
	Time: 3 Months ~ Smoking: Missing	-12.05	3.88	-3.11	0.003	
**Otu00003 *Streptococcus infantis***	Ampicillin	3.92	1.54	2.55	0.014	0.011
	Infant placed on chest immediately: Missing	8.16	3.06	2.67	0.009	
	Breastfeeding: Partial	-3.00	0.70	-4.28	0.000	0.000
	Ethnicity: Malay	-4.77	1.14	-4.19	0.000	0.000
	Ethnicity: Indian	0.02	1.14	0.01	0.989	
	Infant sex: Female	-1.78	0.71	-2.51	0.016	0.012
	Parity	-6.80	2.68	-2.54	0.015	0.011
	Penicillin	3.26	1.02	3.19	0.003	0.001
	Previous breastfeeding experience	7.29	2.71	2.69	0.010	0.007
	Smoking: Some exposure	5.11	2.23	2.29	0.024	
	Time: 3 Months ~ Infant placed on chest immediately: Missing	-9.36	4.00	-2.34	0.023	
	Time: 3 Months ~ Infant placed on chest immediately: No	2.99	1.45	2.06	0.045	0.008
	Time: 3 Months ~ Smoking: Some exposure	-9.60	2.89	-3.32	0.002	
	Time: 3 Months ~ Smoking: No exposure	-8.12	2.64	-3.07	0.003	0.004
**Otu00005**	Infant placed on chest immediately: Missing	4.40	3.32	1.32	0.189	

Klebsiella pneumoniae	Time: 3 Months ~ Augmentin	-5.98	2.20	-2.72	0.009	0.007
	Time: 3 Months ~ Infant placed on chest immediately: Missing	-11.72	3.34	-3.51	0.001	
	Time: 3 Months ~ Infant placed on chest immediately: No	2.90	1.57	1.84	0.071	0.000
	Time: 3 Months ~ Infant sex: Female	-3.00	1.24	-2.43	0.019	0.015
	Time: 3 Months ~ Parity	-13.47	4.90	-2.75	0.008	0.006
	Time: 3 Months ~ Previous breastfeeding experience	16.16	4.90	3.30	0.002	0.001
**Otu00006 *Cutibacterium acnes***	Ethnicity: Malay	-4.75	1.37	-3.46	0.001	0.002
	Ethnicity: Indian	-1.98	1.32	-1.51	0.139	
	Household income: ≥ $6000	2.07	0.83	2.49	0.017	0.040
	Household income: Missing	2.71	3.08	0.88	0.383	
	Pre-pregnancy BMI: Missing	-5.31	1.79	-2.97	0.005	0.005
	Pre-pregnancy BMI: Obese	4.42	2.21	2.00	0.051	
	Pre-pregnancy BMI: Overweight	0.71	0.99	0.72	0.475	
	Pre-pregnancy BMI: Underweight	0.51	1.51	0.34	0.737	
**Otu00007 *Acinetobacter* sp. 2**	Breastfeeding: Partial	2.25	0.88	2.55	0.013	0.011
	Ethnicity: Malay ~ Time: 3 Months	5.98	1.96	3.05	0.004	0.009
	Ethnicity: Indian	-0.73	1.81	-0.40	0.688	
	Ethnicity: Indian ~ Time: 3 Months	0.22	1.87	0.12	0.906	
	Household income: ≥ $6000	-2.91	0.94	-3.09	0.004	0.007
	Household income: Missing	-4.54	3.75	-1.21	0.233	
	Delivery mode: Intrapartum caesarean section	-3.40	1.94	-1.76	0.084	
	Delivery mode: Non-labour caesarean section	-4.68	1.56	-3.00	0.004	0.029
	Maternal age at delivery	0.29	0.11	2.58	0.013	0.010
	Parity	10.72	4.32	2.48	0.017	0.013
	Pre-pregnancy BMI: Missing	-5.13	2.47	-2.08	0.041	0.039
	Pre-pregnancy BMI: Obese	-3.55	2.81	-1.26	0.210	
	Pre-pregnancy BMI: Overweight	-2.12	1.32	-1.60	0.113	
	Pre-pregnancy BMI: Underweight	3.07	1.99	1.54	0.127	
	Previous breastfeeding experience	-10.67	4.29	-2.49	0.017	0.013
	Time: 3 Months ~ Delivery mode: Intrapartum caesarean section	9.84	1.93	5.09	0.000	
	Time: 3 Months ~ Delivery mode: Non-labour caesarean section	3.27	1.66	1.97	0.055	0.000
	Time: 3 Months ~ Pre-pregnancy BMI: Missing	5.70	2.65	2.15	0.037	0.003

	Time: 3 Months ~ Pre-pregnancy BMI: Obese	-4.52	2.74	-1.65	0.105	
	Time: 3 Months ~ Pre-pregnancy BMI: Overweight	-0.48	1.43	-0.33	0.741	
	Time: 3 Months ~ Pre-pregnancy BMI: Underweight	-5.82	2.12	-2.74	0.009	
**Otu00008 *Stenotrophomonas maltophilia***	Breastfeeding: Partial	3.58	0.83	4.30	0.000	0.000
	Ethnicity: Malay	3.68	1.42	2.59	0.012	0.033
	Ethnicity: Indian	0.75	1.33	0.56	0.576	
	Delivery mode: Intrapartum caesarean section	-1.82	1.47	-1.24	0.220	
	Delivery mode: Non-labour caesarean section	-3.03	1.22	-2.49	0.016	0.035
	Time: 3 Months	-1.68	0.61	-2.75	0.008	0.000
	Time: 3 Months ~ Ampicillin	-10.52	2.65	-3.96	0.000	0.000
**Otu00009 *Moraxella osloensis***	Breastfeeding: Partial	2.81	0.94	2.98	0.004	0.003
	Ethnicity: Malay ~ Time: 3 Months	7.47	2.49	3.01	0.004	0.011
	Ethnicity: Indian	1.13	1.89	0.60	0.552	
	Ethnicity: Indian ~ Time: 3 Months	1.34	2.39	0.56	0.578	
	Pre-pregnancy BMI: Obese	4.40	2.75	1.60	0.113	
	Pre-pregnancy BMI: Overweight	-0.88	1.48	-0.59	0.554	
	Pre-pregnancy BMI: Underweight	3.26	2.15	1.52	0.133	
	Time: 3 Months	-1.27	1.12	-1.13	0.263	0.048
	Time: 3 Months ~ Augmentin	-6.27	2.73	-2.30	0.026	0.022
	Time: 3 Months ~ Pre-pregnancy BMI: Missing	4.12	3.40	1.21	0.231	0.007
	Time: 3 Months ~ Pre-pregnancy BMI: Obese	-7.11	3.48	-2.05	0.046	
	Time: 3 Months ~ Pre-pregnancy BMI: Overweight	0.76	1.87	0.41	0.685	
	Time: 3 Months ~ Pre-pregnancy BMI: Underweight	-7.54	2.72	-2.77	0.008	
**Otu00010 *Acinetobacter* sp. 3**	Household income: ≥ $6000	-2.21	0.96	-2.31	0.023	0.032
	Household income: Missing	2.88	3.30	0.87	0.385	
	Pre-pregnancy BMI: Missing	6.58	1.97	3.34	0.001	0.009
	Pre-pregnancy BMI: Obese	-2.62	2.37	-1.11	0.271	
	Pre-pregnancy BMI: Overweight	-0.13	1.05	-0.13	0.899	
	Pre-pregnancy BMI: Underweight	1.34	1.70	0.79	0.434	
	Smoking: Some exposure	-6.07	2.11	-2.87	0.005	
	Smoking: No exposure	-3.72	1.93	-1.93	0.057	0.030
	Smoking: Missing	-3.09	3.09	-1.00	0.321	
**Otu00011 *Streptococcus oralis***	Intrapartum antibiotics	6.50	1.54	4.22	0.000	0.000
	Delivery mode: Intrapartum caesarean section	2.95	1.46	2.02	0.048	
	Delivery mode: Non-labour caesarean section	4.61	1.27	3.64	0.001	0.000

	Parity	-11.31	3.56	-3.18	0.003	0.001
	Previous breastfeeding experience	10.62	3.51	3.03	0.004	0.002
	Time: 3 Months ~ Intrapartum antibiotics	-4.61	1.97	-2.34	0.023	0.020
	Time: 3 Months ~ Breastfeeding: Partial	-3.70	1.55	-2.39	0.020	0.017
	Time: 3 Months ~ Penicillin	9.08	2.64	3.44	0.001	0.001
**Otu00012** ***Gemella haemolysans***	Breastfeeding: Partial	-2.05	0.82	-2.51	0.015	0.012
	Ethnicity: Malay	-4.11	1.38	-2.99	0.004	0.008
	Ethnicity: Indian	0.15	1.29	0.12	0.907	
	Household income: ≥ $6000	2.24	0.91	2.46	0.017	0.045
	Household income: Missing	-1.10	3.20	-0.34	0.733	
	Previous breastfeeding experience	6.45	3.08	2.10	0.041	0.036
	Time: 3 Months ~ Penicillin	5.30	2.08	2.54	0.014	0.011
**Otu00013** ***Bacillus* sp.**	Pre-pregnancy BMI: Missing	6.20	1.56	3.97	0.000	0.001
	Pre-pregnancy BMI: Obese	-1.23	1.56	-0.79	0.433	
	Pre-pregnancy BMI: Overweight	-0.30	0.82	-0.37	0.716	
	Pre-pregnancy BMI: Underweight	0.49	1.24	0.39	0.697	
**Otu00014** ***Acinetobacter* sp. 4**	Infant placed on chest immediately: Missing	-1.39	2.57	-0.54	0.591	0.008
	Breastfed within hours: Yes	-3.19	1.21	-2.64	0.011	0.003
	Breastfeeding: Partial	2.47	0.84	2.94	0.004	0.019
	Ethnicity: Malay ~ Time: 3 Months	5.05	2.35	2.15	0.036	
	Ethnicity: Indian	-2.89	1.75	-1.66	0.101	
	Ethnicity: Indian ~ Time: 3 Months	4.55	2.20	2.07	0.043	
**Otu00015** ***Pseudomonas aeruginosa***	Intrapartum antibiotics	6.97	1.58	4.41	0.000	0.024
	Augmentin	-7.04	2.01	-3.50	0.001	0.042
	Infant placed on chest immediately: Missing	-2.53	1.71	-1.48	0.143	
	Infant placed on chest immediately: No	-1.98	0.82	-2.41	0.018	0.036
	Breastfed within hours: Yes	-2.08	0.82	-2.52	0.013	0.012
	Cephalosporins	14.42	3.26	4.42	0.000	0.008
	Parity	-22.34	4.60	-4.86	0.000	0.004
	Previous breastfeeding experience	23.00	4.69	4.91	0.000	0.001
	Time: 3 Months ~ Intrapartum antibiotics	-8.86	2.21	-4.01	0.000	0.000
	Time: 3 Months ~ Augmentin	8.25	2.83	2.92	0.004	0.004
	Time: 3 Months ~ Cephalosporins	-16.40	4.52	-3.62	0.000	0.000
	Time: 3 Months ~ Parity	25.65	6.49	3.95	0.000	0.000
	Time: 3 Months ~ Penicillin	10.27	2.58	3.98	0.000	0.000
	Time: 3 Months ~ Previous breastfeeding experience	-24.53	6.62	-3.71	0.000	0.000

**Otu00016** ***Pseudomonas* sp.**	Time: 3 Months	1.39	0.64	2.17	0.034	0.030
**Otu00018** ***Rhizobium pusense***	Breastfed within hours: Yes	-3.88	1.23	-3.15	0.003	0.007
	Delivery mode: Intrapartum caesarean section	-2.43	1.47	-1.65	0.105	
	Delivery mode: Non-labour caesarean section	-3.79	1.35	-2.80	0.007	0.013
	Penicillin	-5.25	1.89	-2.77	0.007	0.004
	Time: 3 Months ~ Intrapartum antibiotics	-5.11	1.80	-2.85	0.006	0.004
	Time: 3 Months ~ Penicillin	6.72	2.33	2.89	0.006	
**Otu00020** ***Rothia mucilaginosa***	Intrapartum antibiotics	2.56	1.16	2.22	0.031	0.027
	Breastfed within hours: Missing	5.32	2.61	2.04	0.047	
	Breastfed within hours: Yes	3.19	1.24	2.57	0.013	0.024
	Delivery mode: Intrapartum caesarean section	2.32	1.48	1.57	0.122	
	Delivery mode: Non-labour caesarean section	3.57	1.36	2.62	0.011	0.021
	Time: 3 Months	0.83	0.70	1.18	0.242	0.016
	Time: 3 Months ~ Penicillin	4.64	1.79	2.60	0.012	0.009
**Otu00021** ***Enterobacter hormaechei***	Breastfeeding: Partial	2.89	0.81	3.58	0.001	0.000
	Time: 3 Months ~ Parity	-12.23	4.49	-2.72	0.009	0.007
	Time: 3 Months ~ Previous breastfeeding experience	12.42	4.48	2.77	0.008	0.006
**Otu00023** ***Enterococcus faecalis***	Ampicillin	-11.74	2.97	-3.95	0.000	0.000
	Intrapartum antibiotics	12.40	2.64	4.70	0.000	0.000
	Augmentin	-12.43	2.83	-4.39	0.000	0.000
	Education: University	-1.94	0.72	-2.71	0.009	0.007
	Infant sex: Female	1.60	0.70	2.29	0.027	0.022
	Maternal age at delivery	-0.20	0.08	-2.54	0.014	0.011
	Parity	-14.35	3.71	-3.86	0.000	0.000
	Penicillin	-13.69	2.71	-5.06	0.000	0.000
	Previous breastfeeding experience	12.58	3.65	3.44	0.001	0.001
	Time: 3 Months	-0.91	0.43	-2.13	0.038	0.033
**Otu00024** ***Veillonella dispar***	Infant placed on chest immediately: Missing	1.83	2.88	0.64	0.526	
	Time: 3 Months	0.87	0.95	0.92	0.361	0.027
	Time: 3 Months ~ Ampicillin	8.09	3.72	2.18	0.034	0.029
	Time: 3 Months ~ Intrapartum antibiotics	-5.62	2.22	-2.54	0.014	0.011
	Time: 3 Months ~ Infant placed on chest immediately: Missing	-6.01	3.91	-1.53	0.131	
	Time: 3 Months ~ Infant placed on chest immediately: No	4.07	1.82	2.24	0.029	0.018
	Time: 3 Months ~ Penicillin	9.51	2.76	3.44	0.001	0.001
**Otu00032**	Ampicillin	-11.07	2.38	-4.65	0.000	0.000

Serratia marcescens	Intrapartum antibiotics	11.49	2.09	5.49	0.000	0.000
	Augmentin	-11.02	2.24	-4.92	0.000	0.000
	Parity	-12.37	2.93	-4.22	0.000	0.000
	Penicillin	-11.39	2.17	-5.26	0.000	0.000
	Previous breastfeeding experience	11.78	2.88	4.09	0.000	0.000
	Smoking: Some exposure	-1.62	1.37	-1.19	0.238	
	Smoking: No exposure	-3.54	1.23	-2.87	0.005	0.003
	Smoking: Missing	-4.12	1.88	-2.19	0.030	
	Time: 3 Months	1.41	0.53	2.64	0.010	0.008

Table S3: Outputs of linear mixed effects models for analysis of the full cohort (n=266 samples from 208 mothers). Models were fitted for each response variable. Significant P values are reported. ~ represents an interaction between two variables.

Response	Variable	Estimate	Standard Error	t value	P-value	ANOVA P-value
**Richness**	(Intercept)	3.78	0.05	74.64	0.000	
	Time: 3 Months	0.30	0.08	3.65	0.000	0.011
	Intrapartum antibiotics	0.10	0.09	1.11	0.267	0.584
	Time: 3 Months ~ Intrapartum antibiotics	-0.36	0.13	-2.69	0.008	0.007
**Shannon diversity**	(Intercept)	1.97	0.11	18.20	0.000	
	Time: 3 Months	0.25	0.10	2.42	0.016	0.228
	Education: University	-0.24	0.09	-2.79	0.006	0.017
	Education: Missing	-0.56	0.68	-0.83	0.408	
	Breastfed within hours: Yes	-0.10	0.10	-0.95	0.345	0.023
	Breastfed within hours: Missing	-0.56	0.20	-2.75	0.006	
	Intrapartum antibiotics	0.10	0.11	0.90	0.366	0.494
	Time: 3 Months ~ Intrapartum antibiotics	-0.41	0.17	-2.43	0.016	0.015
**Otu00001 *Staphylococcus epidermidis***	(Intercept)	13.56	0.33	40.65	0.000	
	Time: 3 Months	-0.65	0.28	-2.30	0.023	0.021
	Education: University	0.97	0.32	3.01	0.003	0.006
	Education: Missing	3.03	2.46	1.23	0.219	
	Parity	-0.75	0.32	-2.33	0.021	0.020
	Intrapartum antibiotics	1.20	0.36	3.29	0.001	0.001
	Ampicillin	-1.63	0.62	-2.62	0.009	0.009
**Otu00003 *Streptococcus infantis***	(Intercept)	11.24	0.46	24.57	0.000	
	Ethnicity: Malay	-1.87	0.71	-2.62	0.009	0.031
	Ethnicity: Indian	-0.14	0.74	-0.18	0.855	
	Parity	-3.06	1.42	-2.15	0.032	0.031
	Previous breastfeeding experience	2.92	1.43	2.05	0.042	0.041
	Breastfeeding: Partial	-2.11	0.48	-4.39	0.000	0.000
	Breastfeeding: Missing	-2.35	1.05	-2.25	0.026	
	Ampicillin	2.23	0.86	2.60	0.010	0.009
**Otu00004 *Acinetobacter* sp. 1**	(Intercept)	7.49	0.60	12.53	0.000	
	Ethnicity: Malay	1.63	0.92	1.77	0.078	0.018
	Ethnicity: Indian	-1.71	0.92	-1.85	0.065	
	Time: 3 Months	-1.65	0.76	-2.17	0.032	0.055
	Pre-pregnancy BMI: Missing	-2.17	1.80	-1.20	0.230	0.341
	Pre-pregnancy BMI: Obese	1.56	1.23	1.27	0.206	
	Pre-pregnancy BMI: Overweight	0.44	0.93	0.47	0.635	
	Pre-pregnancy BMI: Underweight	2.08	1.29	1.61	0.108	

	Intrapartum antibiotics	-1.29	0.62	-2.07	0.040	0.039
	Breastfeeding: Partial	1.69	0.60	2.83	0.005	0.010
	Breastfeeding: Missing	2.16	1.28	1.69	0.093	0.013
	Time: 3 Months ~ Pre-pregnancy BMI: Missing	5.14	2.36	2.17	0.031	0.032
	Time: 3 Months ~ Pre-pregnancy BMI: Obese	-4.66	2.57	-1.81	0.072	
	Time: 3 Months ~ Pre-pregnancy BMI: Overweight	2.62	1.45	1.81	0.073	
	Time: 3 Months ~ Pre-pregnancy BMI: Underweight	-1.13	1.92	-0.59	0.556	
**Otu00005** ***Klebsiella pneumoniae***	(Intercept)	7.21	1.06	6.81	0.000	
	Ethnicity: Malay	0.48	1.01	0.47	0.636	0.268
	Ethnicity: Indian	-1.32	1.02	-1.30	0.194	
	Time: 3 Months	-4.76	1.84	-2.58	0.011	0.167
	Household income: ≥ $6000	-0.37	0.73	-0.50	0.615	0.280
	Household income: Missing	-6.35	2.03	-3.13	0.002	
	Infant Sex: Female	1.13	0.69	1.64	0.103	0.500
	Breastfed within hours: Yes	-1.06	0.83	-1.28	0.201	0.744
	Breastfed within hours: Missing	-1.09	1.81	-0.60	0.548	
	Breastfeeding: Partial	0.63	0.71	0.89	0.374	0.021
	Breastfeeding: Missing	1.55	1.25	1.24	0.216	
	Ethnicity: Malay ~ Time: 3 Months	3.12	1.70	1.84	0.068	0.017
	Ethnicity: Indian ~ Time: 3 Months	4.43	1.79	2.47	0.015	
	Time: 3 Months ~ Household income: ≥ $6000	-0.31	1.11	-0.28	0.783	0.010
	Time: 3 Months ~ Household income: Missing	7.95	2.76	2.88	0.004	
	Time: 3 Months ~ Infant Sex: Female	-2.09	1.05	-1.99	0.049	0.047
	Time: 3 Months ~ Breastfed within hours: Yes	3.80	1.38	2.75	0.007	0.018
	Time: 3 Months ~ Breastfed within hours: Missing	1.69	2.57	0.66	0.511	
	Time: 3 Months ~ Breastfeeding: Partial	2.36	1.08	2.18	0.031	0.030
**Otu00006** ***Cutibacterium acnes***	(Intercept)	11.09	2.19	5.06	0.000	
	Ethnicity: Malay	-2.79	0.78	-3.57	0.000	0.001
	Ethnicity: Indian	-0.99	0.80	-1.25	0.213	
	Time: 3 Months	-6.04	2.97	-2.03	0.045	0.506
	Maternal age at delivery	-0.09	0.07	-1.28	0.200	0.752
	Augmentin	2.86	1.06	2.71	0.007	0.007
	Time: 3 Months ~ Maternal age at delivery	0.20	0.09	2.14	0.034	0.032
**Otu00007** ***Acinetobacter* sp. 2**	(Intercept)	5.01	0.58	8.71	0.000	
	Ethnicity: Malay	0.15	0.87	0.18	0.861	0.011
	Ethnicity: Indian	-2.55	0.87	-2.93	0.004	

	Time: 3 Months	-1.39	0.69	-2.02	0.046	0.006
	Pre-pregnancy BMI: Missing	-3.73	1.18	-3.16	0.002	0.010
	Pre-pregnancy BMI: Obese	-0.39	1.08	-0.36	0.717	
	Pre-pregnancy BMI: Overweight	-0.26	0.74	-0.35	0.726	
	Pre-pregnancy BMI: Underweight	1.36	0.97	1.41	0.161	
	Delivery mode: Non-labour caesarean section	-0.42	1.01	-0.42	0.677	0.994
	Delivery mode: Intrapartum caesarean section	-1.44	1.01	-1.42	0.157	
	Intrapartum antibiotics	1.01	0.86	1.17	0.245	0.649
	Breastfeeding: Partial	1.50	0.58	2.59	0.010	0.030
	Breastfeeding: Missing	1.41	1.20	1.18	0.239	
	Penicillin	-0.20	1.18	-0.17	0.863	0.158
	Time: 3 Months ~ Delivery mode: Non-labour caesarean section	1.59	1.53	1.04	0.301	0.016
	Time: 3 Months ~ Delivery mode: Intrapartum caesarean section	4.36	1.57	2.78	0.006	
	Time: 3 Months ~ Intrapartum antibiotics	-3.38	1.25	-2.71	0.007	0.007
	Time: 3 Months ~ Penicillin	3.73	1.66	2.25	0.026	0.025
**Otu00008 *Stenotrophomonas maltophilia***	(Intercept)	-2.63	2.58	-1.02	0.308	
	Ethnicity: Malay	2.21	0.89	2.47	0.014	0.014
	Ethnicity: Indian	-1.00	0.91	-1.10	0.272	
	Time: 3 Months	5.75	3.82	1.51	0.135	0.000
	Maternal age at delivery	0.25	0.08	3.10	0.002	0.020
	Augmentin	-2.63	1.20	-2.20	0.029	0.028
	Time: 3 Months ~ Maternal age at delivery	-0.26	0.12	-2.15	0.033	0.031
**Otu00009 *Moraxella osloensis***	(Intercept)	0.64	2.01	0.32	0.750	
	Time: 3 Months	-1.30	0.56	-2.32	0.021	0.020
	Maternal age at delivery	0.18	0.06	2.91	0.004	0.004
**Otu00010 *Acinetobacter* sp. 3**	(Intercept)	4.37	0.49	8.95	0.000	
	Time: 3 Months	-0.46	0.78	-0.59	0.557	0.231
	Infant Sex: Female	-0.77	0.68	-1.13	0.259	0.869
	Time: 3 Months ~ Infant Sex: Female	2.32	1.12	2.07	0.040	0.039
**Otu00011 *Streptococcus oralis***	(Intercept)	5.40	0.51	10.53	0.000	
	Time: 3 Months	1.12	0.81	1.39	0.167	0.738
	Breastfeeding: Partial	1.08	0.74	1.45	0.149	0.989
	Breastfeeding: Missing	0.40	1.29	0.31	0.755	
	Time: 3 Months ~ Breastfeeding: Partial	-2.59	1.14	-2.26	0.025	0.024
**Otu00012 *Gemella haemolysans***	(Intercept)	5.00	0.46	10.93	0.000	
	Time: 3 Months	-1.44	0.58	-2.50	0.013	0.001

	Education: University	1.74	0.57	3.08	0.002	0.003
	Education: Missing	7.59	4.45	1.71	0.089	
	Ampicillin	3.09	1.00	3.11	0.002	0.002
	Augmentin	5.40	1.61	3.35	0.001	0.027
	Time: 3 Months ~ Augmentin	-5.36	2.12	-2.53	0.013	0.011
**Otu00013** ***Bacillus*** **sp.**	(Intercept)	1.20	1.00	1.20	0.232	
	Time: 3 Months	1.27	2.05	0.62	0.537	0.042
	Infant placed on chest immediately: No	0.66	0.69	0.95	0.343	0.283
	Infant placed on chest immediately: Missing	-4.20	4.00	-1.05	0.294	
	Intrapartum antibiotics	0.12	0.64	0.19	0.852	0.181
	Smoking: No exposure	0.38	1.02	0.38	0.707	0.808
	Smoking: Some exposure	1.15	1.13	1.02	0.309	
	Smoking: Missing	7.15	3.85	1.86	0.065	
	Time: 3 Months ~ Infant placed on chest immediately: No	-2.12	1.12	-1.90	0.060	0.001
	Time: 3 Months ~ Infant placed on chest immediately: Missing	17.77	5.76	3.09	0.002	
	Time: 3 Months ~ Intrapartum antibiotics	-1.97	0.96	-2.06	0.041	0.040
	Time: 3 Months ~ Smoking: No exposure	1.19	2.09	0.57	0.571	0.006
	Time: 3 Months ~ Smoking: Some exposure	0.02	2.27	0.01	0.993	
	Time: 3 Months ~ Smoking: Missing	-18.40	5.56	-3.31	0.001	
**Otu00014** ***Acinetobacter*** **sp. 4**	(Intercept)	1.03	2.24	0.46	0.647	
	Time: 3 Months	-1.27	0.87	-1.46	0.146	0.723
	Maternal age at delivery	0.20	0.06	3.22	0.002	0.001
	Education: University	-2.12	0.57	-3.73	0.000	0.000
	Education: Missing	-7.93	4.41	-1.80	0.073	
	Pre-pregnancy BMI: Missing	1.46	1.65	0.88	0.379	0.059
	Pre-pregnancy BMI: Obese	0.79	1.11	0.71	0.478	
	Pre-pregnancy BMI: Overweight	-0.13	0.81	-0.16	0.870	
	Pre-pregnancy BMI: Underweight	2.73	1.15	2.37	0.018	
	Infant Sex: Female	-0.07	0.64	-0.11	0.910	0.135
	Infant placed on chest immediately: No	-1.99	0.72	-2.75	0.007	0.000
	Infant placed on chest immediately: Missing	-4.90	1.39	-3.53	0.001	
	Breastfed within hours: Yes	-1.85	0.71	-2.60	0.010	0.009
	Time: 3 Months ~ Pre-pregnancy BMI: Missing	0.81	2.19	0.37	0.711	0.025
	Time: 3 Months ~ Pre-pregnancy BMI: Obese	-6.92	2.31	-2.99	0.003	

	Time: 3 Months ~ Pre-pregnancy BMI: Overweight	1.23	1.29	0.95	0.343	
	Time: 3 Months ~ Pre-pregnancy BMI: Underweight	-0.69	1.73	-0.40	0.691	
	Time: 3 Months ~ Infant Sex: Female	2.51	1.03	2.43	0.016	0.015
**Otu00015** ***Pseudomonas aeruginosa***	(Intercept)	-3.00	1.55	-1.94	0.054	
	Time: 3 Months	0.71	0.60	1.17	0.244	0.202
	Maternal age at delivery	0.13	0.05	2.71	0.007	0.007
	Delivery mode: Non-labour caesarean section	-0.40	0.82	-0.48	0.629	0.293
	Delivery mode: Intrapartum caesarean section	2.28	0.81	2.80	0.006	
	Intrapartum antibiotics	1.54	0.58	2.67	0.008	0.081
	Time: 3 Months ~ Delivery mode: Non-labour caesarean section	-0.46	1.37	-0.34	0.735	0.007
	Time: 3 Months ~ Delivery mode: Intrapartum caesarean section	-4.31	1.37	-3.14	0.002	
	Time: 3 Months ~ Intrapartum antibiotics	-1.89	0.91	-2.08	0.039	0.038
**Otu00018** ***Rhizobium pusense***	(Intercept)	-3.04	2.45	-1.24	0.215	
	Time: 3 Months	5.77	3.49	1.65	0.101	0.169
	Maternal age at delivery	0.27	0.07	3.74	0.000	0.002
	Education: University	-1.50	0.54	-2.79	0.006	0.016
	Education: Missing	-3.46	4.14	-0.84	0.404	
	Infant Sex: Female	-0.13	0.62	-0.20	0.838	0.173
	Breastfed within hours: Yes	-1.45	0.65	-2.23	0.027	0.044
	Breastfed within hours: Missing	-2.30	1.24	-1.85	0.065	
	Intrapartum antibiotics	1.12	0.80	1.39	0.165	0.679
	Penicillin	-1.75	1.13	-1.55	0.122	0.693
	Time: 3 Months ~ Maternal age at delivery	-0.22	0.11	-2.00	0.047	0.045
	Time: 3 Months ~ Infant Sex: Female	2.35	0.95	2.48	0.014	0.013
	Time: 3 Months ~ Intrapartum antibiotics	-3.53	1.19	-2.97	0.003	0.003
	Time: 3 Months ~ Penicillin	3.30	1.59	2.08	0.039	0.038
**Otu00020** ***Rothia mucilaginosa***	(Intercept)	3.17	0.33	9.59	0.000	
	Time: 3 Months	1.90	0.50	3.79	0.000	0.000
**Otu00021** ***Enterobacter hormaechei***	(Intercept)	3.77	1.01	3.75	0.000	
	Time: 3 Months	0.68	0.59	1.15	0.253	0.827
	Pre-pregnancy BMI: Missing	0.80	1.54	0.52	0.602	0.551
	Pre-pregnancy BMI: Obese	1.93	1.01	1.90	0.058	
	Pre-pregnancy BMI: Overweight	1.08	0.76	1.43	0.155	
	Pre-pregnancy BMI: Underweight	2.11	1.08	1.97	0.051	

	Smoking: No exposure	-2.01	0.97	-2.08	0.039	0.046
	Smoking: Some exposure	-0.63	1.07	-0.59	0.558	
	Smoking: Missing	-2.09	1.45	-1.45	0.150	
	Breastfeeding: Partial	1.66	0.52	3.19	0.002	0.000
	Breastfeeding: Missing	3.23	1.10	2.95	0.003	
	Time: 3 Months ~ Pre-pregnancy BMI: Missing	-1.40	1.87	-0.75	0.456	0.016
	Time: 3 Months ~ Pre-pregnancy BMI: Obese	-6.38	2.03	-3.14	0.002	
	Time: 3 Months ~ Pre-pregnancy BMI: Overweight	-0.45	1.11	-0.40	0.687	
	Time: 3 Months ~ Pre-pregnancy BMI: Underweight	-2.67	1.51	-1.77	0.079	
**Otu00023** ***Enterococcus faecalis***	(Intercept)	1.72	0.36	4.73	0.000	
	Parity	-1.19	0.46	-2.61	0.010	0.009
	Pre-pregnancy BMI: Missing	0.32	0.95	0.34	0.735	0.004
	Pre-pregnancy BMI: Obese	3.10	0.82	3.80	0.000	
	Pre-pregnancy BMI: Overweight	0.88	0.58	1.50	0.135	
	Pre-pregnancy BMI: Underweight	0.07	0.77	0.09	0.928	
**Otu00024** ***Veillonella dispar***	(Intercept)	4.26	0.60	7.12	0.000	
	Time: 3 Months	0.53	1.05	0.50	0.617	0.000
	Household income: ≥ $6000	-0.17	0.66	-0.25	0.803	0.405
	Household income: Missing	-2.50	1.95	-1.28	0.202	
	Delivery mode: Non-labour caesarean section	-0.45	0.99	-0.46	0.647	0.960
	Delivery mode: Intrapartum caesarean section	-1.78	1.02	-1.75	0.081	
	Previous breastfeeding experience	-1.03	0.66	-1.56	0.121	0.948
	Time: 3 Months ~ Household income: ≥ $6000	-1.34	1.11	-1.21	0.229	0.045
	Time: 3 Months ~ Household income: Missing	4.79	2.59	1.85	0.066	
	Time: 3 Months ~ Delivery mode: Non-labour caesarean section	0.83	1.65	0.50	0.617	0.026
	Time: 3 Months ~ Delivery mode: Intrapartum caesarean section	4.61	1.71	2.70	0.008	
	Time: 3 Months ~ Previous breastfeeding experience	3.00	1.09	2.74	0.007	0.006
**Otu00032** ***Serratia marcescens***	(Intercept)	0.23	0.27	0.84	0.401	
	Ethnicity: Malay	1.08	0.53	2.06	0.041	0.022
	Ethnicity: Indian	1.17	0.54	2.15	0.033	
	Time: 3 Months	1.10	0.38	2.89	0.004	0.033
	Intrapartum antibiotics	2.99	1.08	2.78	0.006	0.005
	Penicillin	-1.21	1.19	-1.02	0.308	0.035
	Cephalosporins	-2.75	1.15	-2.38	0.018	0.017
	Ampicillin	-2.86	1.21	-2.36	0.019	0.019

Augmentin	-3.12	1.25	-2.49	0.013	0.013
Time: 3 Months ~ Penicillin	-2.22	0.95	-2.33	0.021	0.020

Table S4: Taxa included in the oral bacteria model. References to evidence for these taxa as members of the human oral microbiome (and the infant oral microbiome, where available) are provided.

Species	Relative abundance (%)	Reference
Streptococcus infantis	8.14	(44, 113, 114)
Streptococcus salavarius	7.48	(43, 113-118)
Klebsiella pneumoniae	3.68	(119-121)
Streptococcus oralis	1.85	(114, 118, 122)
Gemella haemolysans	1.68	(43, 114, 116, 118, 123)
Pseudomonas sp.	1.02	(121, 124-126)
Pseudomonas aeruginosa	0.87	(121, 124)
Rothia mucilaginosa	0.65	(43, 114, 116-118)
Veillonella dispar	0.65	(116, 117, 127, 128)

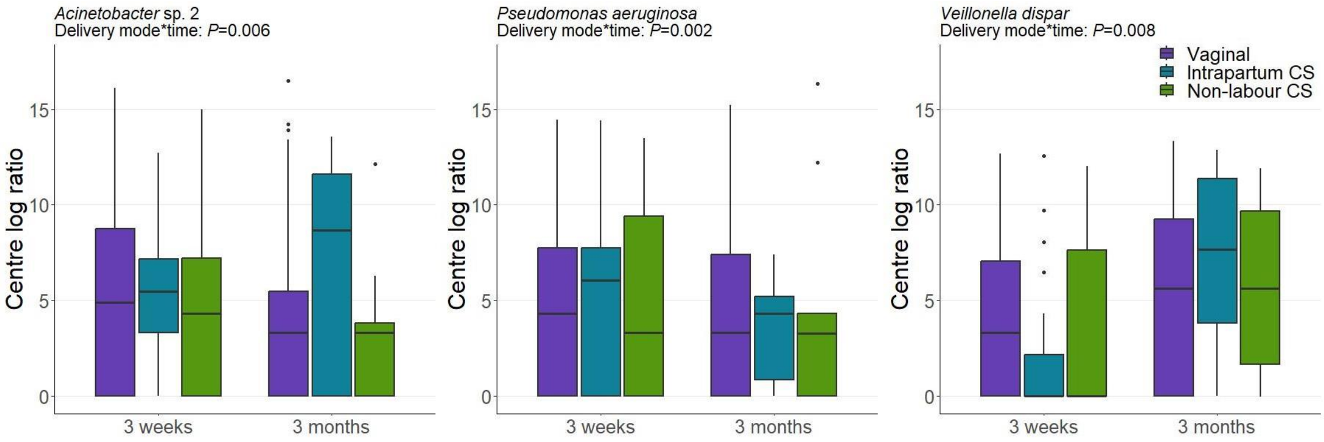

Figure S1: The interaction between delivery mode and time was significantly associated with three taxa of the human milk microbiome. Data are CLR transformed abundance. ANOVA *P* values derived from linear mixed effects models are displayed.

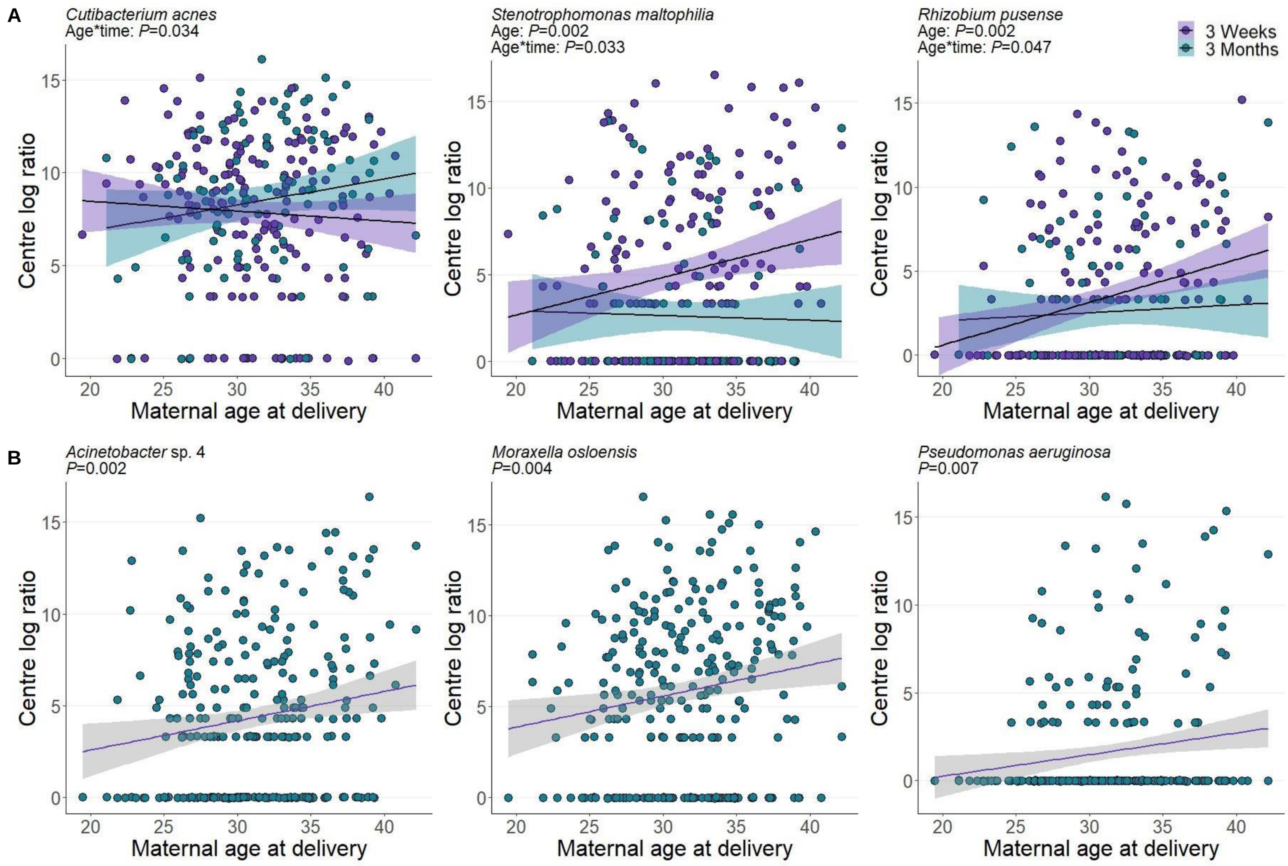

Figure S2: (A) Associations between maternal age at delivery and taxa of the human milk microbiome when interacted with time. (B) Associations between maternal age at delivery and taxa of the human milk microbiome. Data are CLR transformed abundance. *P* values are derived from linear mixed effects models.

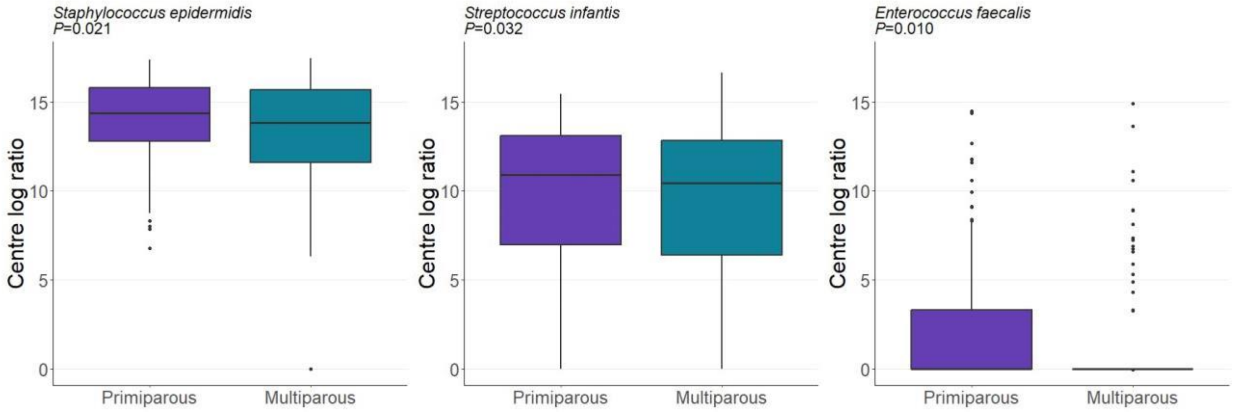

Figure S3: Associations between parity and taxa of the human milk microbiome. Data are CLR transformed abundance. *P* values are derived from linear mixed effects models.

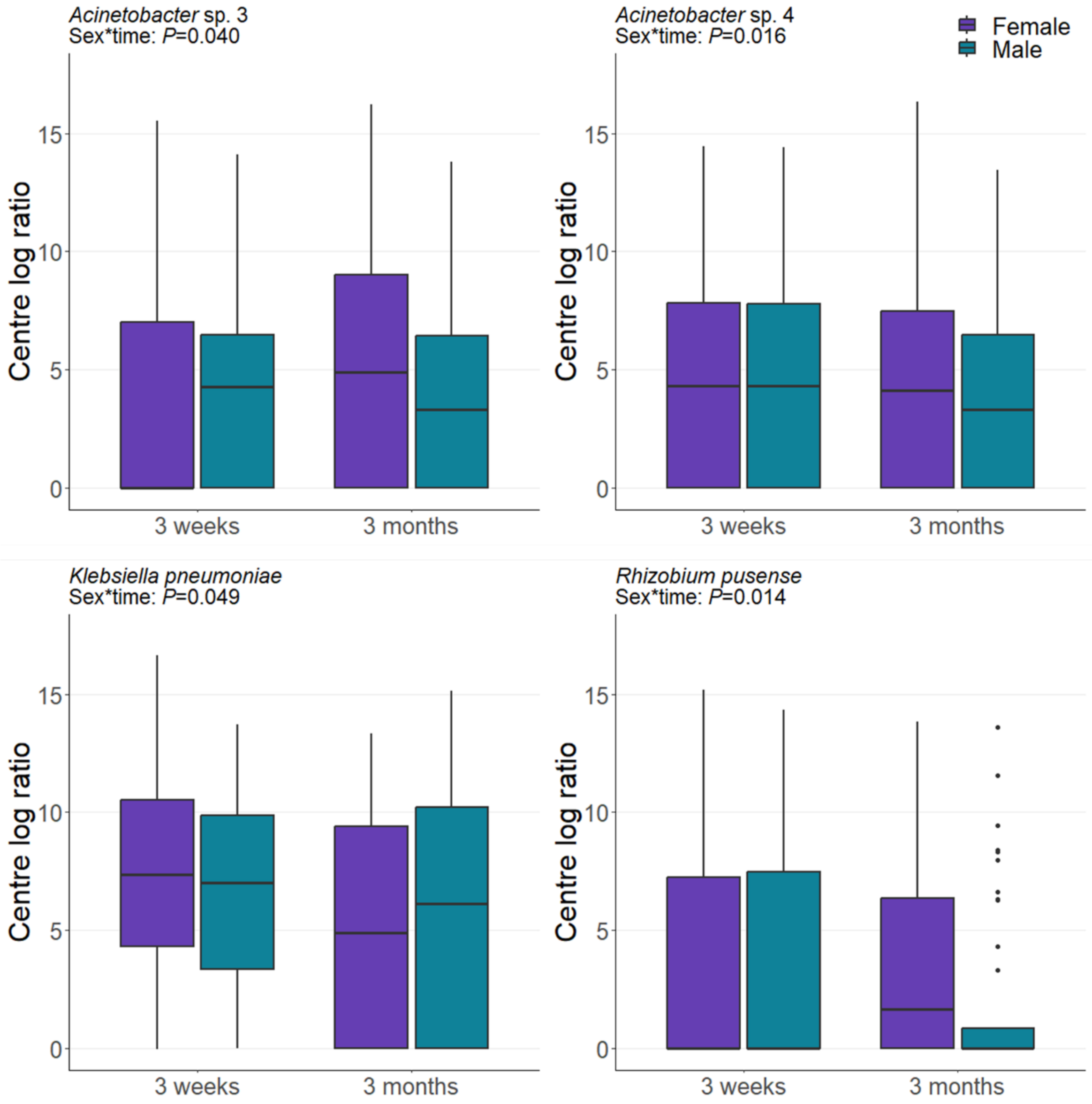

Figure S4: The interaction between infant sex and time was significantly associated with three taxa of the human milk microbiome. Data are CLR transformed abundance. *P* values are derived from linear mixed effects models.

REFERENCES

43. Cheema AS, Trevenen ML, Turlach BA, Furst AJ, Roman AS, Bode L, Gridneva Z, Lai CT, Stinson LF, Payne MS, Geddes DT. 2022. Exclusively breastfed infant microbiota develops over time and is associated with human milk oligosaccharide intakes. Int J Mol Sci 23:2804. https://doi.org/10.3390/ijms23052804

44. Biagi E, Quercia S, Aceti A, Beghetti I, Rampelli S, Turroni S, Faldella G, Candela M, Brigidi P, Corvaglia L. 2017. The bacterial ecosystem of mother's milk and infant's mouth and gut. Front Microbiol 8:1214. https://doi.org/10.3389/fmicb.2017.01214

113. Dzidic M, Collado MC, Abrahamsson T, Artacho A, Stensson M, Jenmalm MC, Mira A. 2018. Oral microbiome development during childhood: an ecological succession influenced by postnatal factors and associated with tooth decay. ISME J 12:2292–2306. https://doi.org/10.1038/s41396-018-0204-z

114. Sulyanto RM, Thompson ZA, Beall CJ, Leys EJ, Griffen AL. 2019. The pPredominant oral microbiota is acquired early in an organized pattern. Sci Rep 9:10550. https://doi.org/10.1038/s41598-019-46923-0

115. Carlsson J, Grahnén H, Jonsson G, Wikner S. 1970. Early establishment *Streptococcus salivarius* in the mouth of

infants. J Dent Res 49:415–418. https://doi.org/10.1177/00220345700490023601

116. Butler CA, Adams GG, Blum J, Byrne SJ, Carpenter L, Gussy MG, Calache H, Catmull DV, Reynolds EC, Dashper SG. 2022. Breastmilk influences development and composition of the oral microbiome. J Oral Microbiol 14:2096287. https://doi.org/10.1080/20002297.2022.2096287

117. Zhang Y, Wu YP, Feng V, Cao GZ, Feng XP, Chen X. 2022. Microbiota of preterm infant develops over time along with the first teeth eruption. Front Microbiol 13:1049021. https://doi.org/10.3389/fmicb.2022.1049021

118. Dashper SG, Mitchell HL, Lê Cao K-A, Carpenter L, Gussy MG, Calache H, Gladman SL, Bulach DM, Hoffmann B, Catmull DV, Pruilh S, Johnson S, Gibbs L, Amezdroz E, Bhatnagar U, Seemann T, Mnatzaganian G, Manton DJ, Reynolds EC. 2019. Temporal development of the oral microbiome and prediction of early childhood caries. Sci Rep 9:19732. https://doi.org/10.1038/s41598-019-56233-0

119. Baker JL, Hendrickson EL, Tang X, Lux R, He X, Edlund A, McLean JS, Shi W. 2019. *Klebsiella* and *Providencia* emerge as lone survivors following long-term starvation of oral microbiota. Proc Natl Acad Sci USA 116:8499–8504. https://doi.org/10.1073/pnas.1820594116

120. Sumi Y, Miura H, Michiwaki Y, Nagaosa S, Nagaya M. 2007. Colonization of dental plaque by respiratory pathogens in dependent elderly. Arch Gerontol Geriatr 44:119–124. https://doi.org/10.1016/j.archger.2006.04.004

121. Leão I, de Carvalho TB, Henriques V, Ferreira C, Sampaio-Maia B, Manaia CM. 2023. Pseudomonadota in the oral cavity: a glimpse into the environment-human nexus. Appl Microbiol Biotechnol 107:517–534. https://doi.org/10.1007/s00253-022-12333-y

122. Okahashi N, Nakata M, Kuwata H, Kawabata S. 2022. Oral mitis group streptococci: A silent majority in our oral cavity. Microbiol Immunol 66:539–551. https://doi.org/10.1111/1348-0421.13028

123. Miyoshi T, Oge S, Nakata S, Ueno Y, Ukita H, Kousaka R, Miura Y, Yoshinari N, Yoshida A. 2021. Gemella haemolysans inhibits the growth of the periodontal pathogen porphyromonas gingivalis. Sci Rep 11:11742. https://doi.org/10.1038/s41598-021-91267-3

124. Dewhirst FE, Chen T, Izard J, Paster BJ, Tanner ACR, Yu W-H, Lakshmanan A, Wade WG. 2010. The human oral microbiome. J Bacteriol 192:5002–5017. https://doi.org/10.1128/JB.00542-10

125. Xu H, Li X, Zheng X, Xia Y, Fu Y, Li X, Qian Y, Zou J, Zhao A, Guan J, Gu M, Yi H, Jia W, Yin S. 2018. Pediatric obstructive sleep apnea is associated with changes in the oral microbiome and urinary metabolomics profile: A pilot study. J Clin Sleep Med 14:1559–1567. https://doi.org/10.5664/jcsm.7336

126. Willis JR, Saus E, Iraola-Guzmán S, Cabello-Yeves E, Ksiezopolska E, Cozzuto L, Bejarano LA, Andreu-Somavilla N, Alloza-Trabado M, Blanco A, Puig-Sola A, Broglio E, Carolis C, Ponomarenko J, Hecht J, Gabaldón T. 2021. Citizen-science based study of the oral microbiome in Cystic fibrosis and matched controls reveals major differences in diversity and abundance of bacterial and fungal species. J Oral Microbiol 13:1897328. https://doi.org/10.1080/20002297.2021.1897328

127. Kageyama S, Furuta M, Takeshita T, Ma J, Asakawa M, Yamashita Y. 2022. High-level acquisition of maternal oral bacteria in formula-fed infant oral microbiota. mMBio 13:e0345221. https://doi.org/10.1128/mbio.03452-21

128. Djais AA, Theodorea CF, Mashima I, Otomo M, Saitoh M, Nakazawa F. 2019. Identification and phylogenetic analysis of oral veillonella species isolated from the saliva of Japanese children. F1000Res 8:616. https://doi.org/10.12688/f1000research.18506.5
